# Peroxisomal biogenesis is genetically and biochemically linked to carbohydrate metabolism in Drosophila and mouse

**DOI:** 10.1371/journal.pgen.1006825

**Published:** 2017-06-22

**Authors:** Michael F. Wangler, Yu-Hsin Chao, Vafa Bayat, Nikolaos Giagtzoglou, Abhijit Babaji Shinde, Nagireddy Putluri, Cristian Coarfa, Taraka Donti, Brett H. Graham, Joseph E. Faust, James A. McNew, Ann Moser, Marco Sardiello, Myriam Baes, Hugo J. Bellen

**Affiliations:** 1 Department of Molecular and Human Genetics, Baylor College of Medicine (BCM), Houston, TX, United States of America; 2 Texas Children’s Hospital, Houston TX, United States of America; 3 Program in Developmental Biology, BCM, Houston, TX, United States of America; 4 Jan and Dan Duncan Neurological Research Institute, Texas Children’s Hospital (TCH), Houston, TX, United States of America; 5 KU Leuven, Laboratory of Cell Metabolism, Department of Pharmaceutical and Pharmacological Sciences, Leuven, Belgium; 6 Department of Molecular and Cellular Biology, BCM, Houston, TX, United States of America; 7 Department of BioSciences, Rice University, Houston TX, United States of America; 8 Kennedy Krieger Institute, Baltimore MD, United States of America; 9 Howard Hughes Medical Institute, Houston, TX, United States of America; 10 Department of Neuroscience, BCM, Houston, TX, United States of America; University of Washington, UNITED STATES

## Abstract

Peroxisome biogenesis disorders (PBD) are a group of multi-system human diseases due to mutations in the *PEX* genes that are responsible for peroxisome assembly and function. These disorders lead to global defects in peroxisomal function and result in severe brain, liver, bone and kidney disease. In order to study their pathogenesis we undertook a systematic genetic and biochemical study of Drosophila *pex16* and *pex2* mutants. These mutants are short-lived with defects in locomotion and activity. Moreover these mutants exhibit severe morphologic and functional peroxisomal defects. Using metabolomics we uncovered defects in multiple biochemical pathways including defects outside the canonical specialized lipid pathways performed by peroxisomal enzymes. These included unanticipated changes in metabolites in glycolysis, glycogen metabolism, and the pentose phosphate pathway, carbohydrate metabolic pathways that do not utilize known peroxisomal enzymes. In addition, mutant flies are starvation sensitive and are very sensitive to glucose deprivation exhibiting dramatic shortening of lifespan and hyperactivity on low-sugar food. We use bioinformatic transcriptional profiling to examine gene co-regulation between peroxisomal genes and other metabolic pathways and we observe that the expression of peroxisomal and carbohydrate pathway genes in flies and mouse are tightly correlated. Indeed key steps in carbohydrate metabolism were found to be strongly co-regulated with peroxisomal genes in flies and mice. Moreover mice lacking peroxisomes exhibit defective carbohydrate metabolism at the same key steps in carbohydrate breakdown. Our data indicate an unexpected link between these two metabolic processes and suggest metabolism of carbohydrates could be a new therapeutic target for patients with PBD.

## Author summary

Peroxisomes are organelles or component of cells that are involved in body chemistry for a number of specialized fats. Peroxisome biogenesis disorders (PBD) are a group of rare diseases in which patients have genetic defects in the synthesis of peroxisomes. These disorders affect multiple organs including the brain and liver. We used fruitfly (*Drosophila* melanogaster) to study the metabolism and genetics of peroxisomal biogenesis to gain insight into the disease process. We generated flies with genetic defects in peroxisomes, carefully characterized these flies finding that they are short-lived and have locomotor problems. We then applied global metabolic profiling in these flies, measuring hundreds of biochemical compounds. The analysis pointed to an unexpected link between peroxisomes and sugar metabolism. Guided by this we found our flies were sensitive to low-sugar diet. We then used gene-expression analysis and targeted biochemical profiling in mouse to confirm that carbohydrate alterations also occur in vertebrates. Our work suggests carbohydrate metabolism may be a crucial process to study in patients with PBD.

## Introduction

Peroxisomes are ubiquitous organelles present in all eukaryotic cells [1–3]. Peroxisomes perform specific biochemical functions in the cell including fatty acid β-oxidation of very-long-chain fatty acids (VLCFA) [4], α-oxidation of branched chain fatty acids [5, 6], plasmalogen biosynthesis [7, 8], and also participate in the metabolism of reactive oxygen species [9] and glyoxylate [10, 11]. Peroxisomes are formed by the action of 14 peroxins encoded by *PEX* genes, the majority of which are involved in translocation of peroxisomal enzymes into the matrix, with others designating peroxisomal membrane [12–15]. Human diseases due to autosomal recessive loss of function mutations in the *PEX* genes comprise a group of severe disorders known as peroxisome biogenesis disorders (PBD) with involvement of brain, bone, kidney and liver and death within the first year of life [1, 2, 16, 17].

The peroxisome’s well documented role in β-oxidation of VLCFA and synthesis of ether lipids has led to considerable focus on lipid metabolism as the key pathogenic factor in disease pathogenesis in PBD [18]. The accumulation of VLCFA has been proposed as the primary pathway influencing severity and as a therapeutic target [19–21]. A more general alteration of peroxisomal lipids have been proposed as a developmental insult to the brain in PBD [22].

However, while the increases in VLCFA and loss of plasmalogens in peroxisomal metabolism are likely to be a significant part of the pathogenesis of PBD, other metabolic pathways are also likely to play a role. Indeed, patients with pathogenic variants in *PEX2* [23, 24], *PEX10* [25] and *PEX16* [26, 27] that allow survival into childhood or adulthood have been reported with very mild abnormalities in VLCFA metabolism, and plasmalogen biosynthesis. These studies suggest that additional or even distinct peroxisomal functions are involved in PBD pathogenesis.

Peroxisomal biology is highly conserved across eukaryotes which has allowed this same genetic machinery to be studied across several model organisms [28]. In mice, studies of a spectrum of enzymatic and biogenesis defects in global and conditional knockouts has allowed insight into the role of peroxisomes in vertebrate tissues [29]. Severe early phenotypes affecting brain, growth, and viability have been observed in *Pex2*, *Pex5* and *Pex13* knock-out mice [30–32]. In addition a *Pex1* knock-in for a common missense allele in human PBD produces mice with growth failure, cholestasis and retinopathy [33]. *Pex* genes have been shown to have tissue specific effects. For example, an oligodendrocyte-specific loss of peroxisomal biogenesis produces much of the axonal loss and demyelination seen in PBD suggesting a cell autonomous role of peroxisomes in oligodendrocytes [34]. Hepatocyte knockouts produce effects on mitochondrial morphology and ER stress [35–37]. Several recent studies have also explored peroxisomal biogenesis in *Drosophila* demonstrating the evolutionary conservation [38–42]. Studies of *Drosophila pex* mutants demonstrated a role for VLCFA in interfering with spermatogenesis leading to infertility [42]. In addition, fly *pex16* mutants have been shown to have locomotor defects, and shortened lifespan [41]. Collectively, the study of peroxisomes in flies and mice have provided compelling data that the function of peroxisomes in longevity, locomotion and metabolism are conserved from flies to man.

A key question that has not been addressed by the previous fly studies is whether the phenotype due to loss of peroxisomes is determined by any pathways in metabolism beyond peroxisomal lipids. Indeed, a comprehensive metabolic profile of peroxisomal biogenesis mutants is lacking. Here we utilize genetics, transcriptional informatics and untargeted metabolomics to show that *Drosophila pex* mutants exhibit an unanticipated defect in sugar metabolism and are sensitive to reduced dietary sugar. We also find a strong transcriptional co-regulation between peroxisomal genes in the fly and enzymes in glucose metabolism, and show that similar transcriptional signatures are observed in mice.

## Results

### Genetic tools for peroxisomal biogenesis studies in *Drosophila pex2* and *pex16*

**Fig 1:**
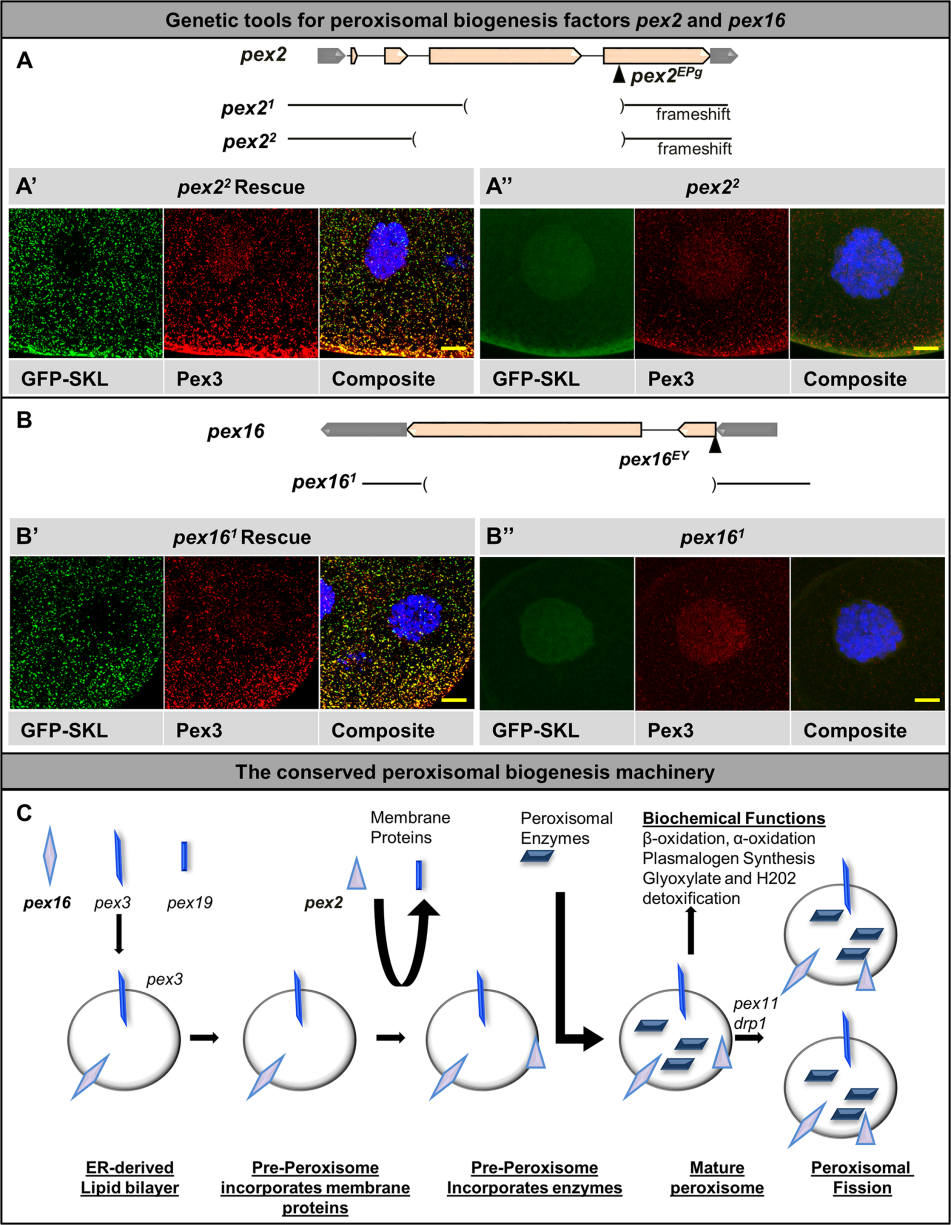
Genetic tools for studies of peroxisomal biogenesis in *Drosophila*. A) Genetic tools to study *pex2*. Two deletion alleles were produced by imprecise excision of an EPg *P*-element (shown to scale, see Materials and Methods) for the *pex2* gene. These alleles were crossed to molecularly characterized deficiency strains for all experiments (shown to scale, see Materials and Methods). (A’) Confocal microscopy image of *pex2*^*2*^ with the P[acman] genomic rescue construct (*pex2*^*2*^ Rescue). Salivary gland peroxisomes were imaged under confocal microscopy. The GFP-SKL marker (green) demonstrating punctate localization, these punctae co-localize with the Pex3 antibody stain (red), error bar 10 μm. (DAPI is shown in the composite) (A”) Confocal microscopy image of the *pex2*^*2*^ mutants. These mutants exhibit loss of the punctate staining, and some nuclear localization is observed. DAPI labels nuclei. Scale bar 10 μm. (DAPI is shown in the composite) (B) Genetic tools to study *pex16*. The *pex16* gene is shown in genomic context, a deletion allele [41] and an EY *P*-element strain were used. In addition, a P[acman] genomic rescue strain was used to generate rescue strains. (B’) Confocal microscopy image of *pex16*^*1*^ with the P[acman] genomic rescue construct (*pex16*^*1*^ Rescue). Salivary gland peroxisomes demonstrate a similar punctate localization of GFP-SKL and Pex3 with good co-localization. (B”) *pex16*^*1*^ mutants display a similar loss of punctae with both GFP-SKL and Pex3 antibody stain. DAPI labels nuclei. Scale bar 10 μm. (DAPI is shown in the composite) (C) The conserved peroxisomal biogenesis machinery is shown in schematic. Early peroxisomal proteins, pex3, pex19 and pex16 (purple diamond for pex16 and blue symbols) aid in designation of an ER-derived lipid bilayer (left). Membrane proteins are then incorporated, and enzymes (dark blue rhomboids) can subsequently be imported into the maturing peroxisome (black block arrows). This process requires *pex2*, a component of the importomer complex (purple triangle). Mature peroxisomes perform a range of biochemical functions in lipid oxidation and detoxification. Mature peroxisomes can then undergo fission.

To perform detailed phenotypic and biochemical analysis and identify new pathways that may be affected when peroxisomal function is lost, we studied two genes required for *Drosophila* peroxisomal biogenesis. We selected *pex16* and *pex2* because both are conserved PBD disease gene homologs that act in distinct steps of peroxisomal biogenesis (**Fig 1**) ensuring that our results will uncover key, cell biological aspects of peroxisomes rather than gene specific ones. While *pex16* is involved in early peroxisomal membrane formation, *pex2* is a component of an ubiquitination complex that functions in matrix protein import [43]. In order to study loss-of-function mutants for *pex2* we obtained an EPg element [44], a *P*-element insertion in the fourth coding exon of the *pex2* gene (**Fig 1A**). However, this allele has not been characterized in detail: it is not known if it corresponds to a null allele, nor have the phenotypes been rescued with a transgene. We therefore performed imprecise excision and produced two additional loss-of-function alleles (*pex2*^*1*^, a 473 bp deletion and *pex2*^*2*^, a 599 bp deletion). For *pex16* we studied a EPgy2 *P*-element insertion allele [45] in the 5’UTR of the *pex16* gene which behaves as a hypomorphic allele [42](**Fig 1B**). We also obtained a *pex16* deletion mutant that lacks the coding region of the gene [41]. For each strain we studied the mutant alleles in trans with a genomic deficiency and used genomic rescue constructs to rescue the phenotypes and to ascertain that the observed phenotypes are not due to second site hits (**S1 Fig**, see Materials and Methods). To investigate the peroxisomal phenotypes we examined the mutant larval salivary glands using the Pex3 antibody [38] and a peroxisomally localized green fluorescent protein (GFP-SKL) (**Fig 1A’**). The UAS-GFP-SKL construct was driven by the ubiquitous strong driver Act5C-GAL4. GFP-SKL produces a GFP with PTS1 targeting signal for the peroxisomal biogenesis machinery encoded by the *pex* genes necessary to import the protein into the peroxisomal matrix [46]. In *Drosophila*, PTS1 is the only system that allows localization into the peroxisomal matrix as PTS2 proteins are not present[39]. In control and rescued animals we observed microscopic punctae in which GFP-SKL and Pex3 extensively co-localize (**Fig 1A’ and 1A”** rescue shown). However, *pex2* mutant cells exhibited a mostly nuclear distribution of GFP-SKL and severely reduced Pex3 staining (**Fig 1A’ and 1A”**). Similarly, in *pex16* mutants we observe diffuse localization of GFP-SKL and reduced Pex3 signal compared to rescue (**Fig 1B’ and 1B”**). Our data suggest that loss of either gene causes defective localization of peroxisomal markers and hence are likely to disrupt peroxisomal biogenesis/function. Therefore these genetic tools permit comparison of *pex2* and *pex16* mutants, two mutants that represent defects in different steps of the peroxisomal biogenesis pathway (**Fig 1C**).

### *Drosophila pex2* and *pex16* mutants exhibit peroxisomal dysfunction consistent with PBD

**Fig 2:**
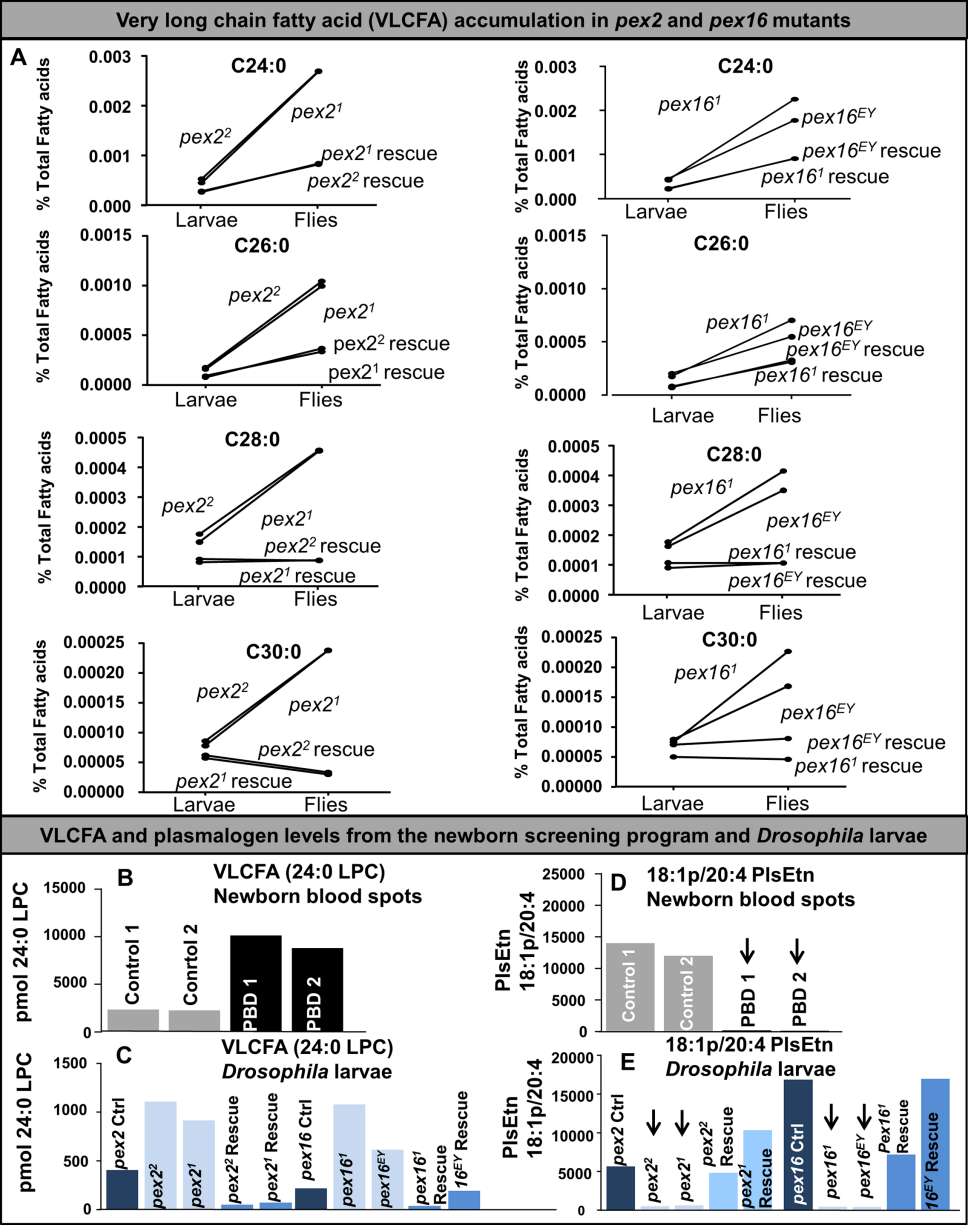
Very-long chain fatty acid (VLCFA) accumulation and plasmalogen deficiency in *Drosophila pex* mutants. (A) Third instar larvae and adult flies of *pex2*^*2*^ and *pex2*^*1*^ mutants, *pex16*^*1*^ and *pex16*^*EY*^ mutants, as well as each rescue were flash frozen in liquid nitrogen and stored at -80°C. These were analyzed by Gas chromatography mass spectrometry (see methods) for C24:0, C26:0, C28:0 and C30:0. Ten larvae and ten flies were analyzed for each genotype. These analyses revealed different abundance for larvae versus flies for each lipid consistent with distinct stages in development. Results of these analyses revealed a higher level for all four metabolites in larvae of all the *pex* mutant alleles. There was a substantial increase in adult flies for the mutants for all of these analytes, while rescue exhibited either decreased, or minimal increase in C24:0 and C26:0. (B) VLCFA analysis on individual newborn blood spots using LC-MSMS, measurement of pmol of 24:0 LPC per blood spot is shown for control, and two PBD infants. (C) VLCFA analysis on individual *Drosophila* larvae (pooled sample of 10 larvae each) using the LC-MSMS, measurements of pmol of 24:0LPC for 10 larvae are shown. (D) LC-MSMS measurements of 18:1p/20:4 ethanolamine plasmalogen, (PlsEtn), in newborn blood spots. As expected the samples from infants with PBD have dramatic loss of plasmalogen resulting in low level compared to controls. (E) LC-MSMS measurements of 18:1p/20:4 ethanolamine plasmalogen, (PlsEtn) with individual measurements of 10 *Drosophila* larvae from each genotype. The peroxisomal mutant flies exhibit a dramatic reduction in the level suggesting plasmalogen loss.

To assess if peroxisomal function was altered in our mutants we examined canonical peroxisomal lipids using the same methods as employed for clinical diagnosis [47–49]. We used Gas chromatograph mass spectrometry (GC-MS) to measure very long chain fatty acids in larvae and whole adult flies [49] and observed only mildly increased levels of C30:0, C28:0, C26:0 and C24:0 in larval *pex2* mutants as well as *pex16* mutants (**Fig 2A**). However, we observed a dramatic increase in these lipids in adult flies compared to larvae in the mutants (**Fig 2A)**.

We also measured the levels of lyso-phosphatidylcholines (LPC), C16:0 to C26:0 LPCs, and the 4 phosphatidylethanolamine plasmalogen (PlsEtn) species (16:0p/20:4, 16:0p/18:1, 18:1p/20:4 and 18:0p/20:4) in third instar larvae and found increased 24:0 LPC levels and decreased 18:1p/20:4 PlsEtn levels by LC-MSMS [47, 48, 50] (**Fig 2C and 2E, S1 Table**). These assays were conducted along with human samples from controls, and patients with PBD (**Fig 2B and 2D, S1 Table**). We noted a dramatic difference between the mutant animals compared to control and rescue (**Fig 2C and 2E, S1 Table**). The 24:0 LPC accumulates quite dramatically concomitant with a severe loss of plasmalogen in both the *pex2* and the *pex16* mutant larvae, consistent with a defect in VLCFA catabolism and plasmalogen synthesis, defects that are seen in PBD. However, interestingly these biochemical defects are distinct from those observed in patients with PBD as the C26:0 LPC was not increased in the mutant larvae whereas in humans with PBD C26:0 LPC shows the greatest increase when compared to controls (**S1 Table**). In addition, in PBD levels of all 4 PlsEtn are typically reduced. These data indicate that a fundamental role for peroxisomes in VLCFA catabolism and plasmalogen synthesis is conserved but some of the specific lipids affected differ between flies and humans.

### *Drosophila pex2* and *pex16* mutants are short lived, bang-sensitive and have locomotor defects

**Fig 3:**
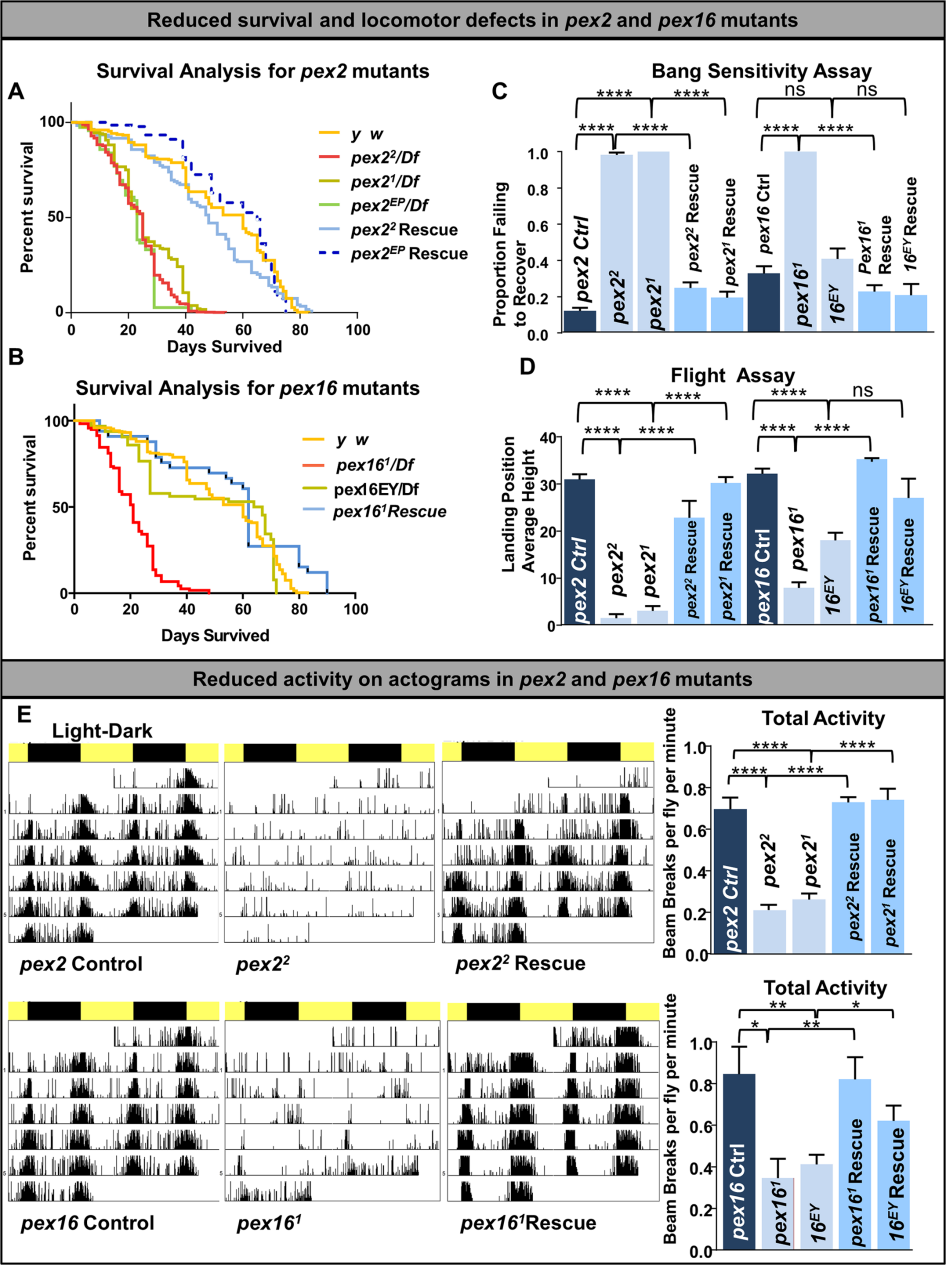
Reduced survival and locomotion defects in *pex2* and *pex16* mutants. (A) Survival curves on standard media at room temperature for *pex2* mutant, control and rescue strains. Color codes show the genotypes with *y w* flies (yellow), *pex2*^*2*^ Rescue (solid blue), and *pex2*^*EP*^ rescue (blue dotted) living up to 80 days, and *pex2*^*1*^*/Df*,(dark green) *pex2*^*2*^*/Df* (red) and *pex2*^*EP*^*/Df* (green) with shortened lifespan. (B) Survival curves on standard media at room temperature for *pex16* mutant, control and rescue strains. Color codes show the genotypes with *y w* flies (yellow), *pex16*^*1*^*/Df* (red) and *pex16*^*1*^rescue (blue). (C) Bang sensitivity assay in *pex* mutant flies. Flies of the 10 indicated genotypes were subjected in vials to vortex for 10 seconds and timed to how long they took to recover. Shown is the proportion failing to recover after 10 seconds. For the data shown the number of flies for each trial was *pex2* Ctrl (26), *pex2*^*2*^*/Df* (8), *pex2*^*1*^*/Df* (6), *pex2*^*2*^ Rescue (14), *pex2*^*1*^ Rescue (18) *pex16 Ctrl* (34) *pex16*^*1*^*/Df (16) pex16*^*EY*^*/Df (25) pex16*^*1*^ Rescue (18) *pex16*^*EY*^ Rescue (39) (D) Flight assay was performed in 10 day old flies. Flies of the 10 indicated genotypes were lightly tapped into a clear column made of PVC marked at each centimeter. The average height of their landing position on the column is shown. For the data shown the number of flies for each trial was *pex2* Ctrl (97), *pex2*^*2*^*/Df* (65), *pex2*^*1*^*/Df* (58), *pex2*^*2*^ Rescue (21), *pex2*^*1*^ Rescue (86) *pex16 Ctrl* (131) *pex16*^*1*^*/Df (143) pex16*^*EY*^*/Df (101) pex16*^*1*^ Rescue (95) *pex16*^*EY*^ Rescue (13) (E) *Drosophila* activity monitor with reduced activity in 1–2 day old *pex2* and *pex16* mutant flies. The *Drosophila* activity monitoring actograms are shown with 12 hour light/dark cycles depicted by yellow/dark bars across the top of each actogram. Representative actograms for single flies for each indicated genotype are shown. The y-axis of each actrogram shows the number of beam breaks per minute and the total activity in beam breaks per fly per minute is shown at right.

We next explored the phenotypes of the *pex* mutant flies. *Drosophila pex2* mutants have been reported to have spermatogenesis defects without appreciable nervous system phenotypes [42], while *pex16* mutants have been noted to have shortened survival and locomotor defects [41]. Given the difference in reported phenotypes, we undertook an extensive phenotypic characterization of both *pex2* and *pex16* mutants. We noted that the survival of both *pex2* (**Fig 3A**) and *pex16*^*1*^mutants (**Fig 3B**) is dramatically shortened to approximately 50% of controls. For the *pex16*^*EY*^ mutants we observed intermediate survival consistent with this being a hypomorphic allele (**Fig 3B**).We also observed that young (2–3 day) flies performed poorly in a bang sensitivity assay in which they were subject to mechanical stress and allowed to recover (**Fig 3C, S1 Video (***pex2* control at left, *pex2*^*2*^ at center and *pex2*^*2*^ Rescue at right; **S2 Video** p*ex16* control at left, *pex16*^*1*^at center, and *pex16^1^* Rescue at right). Moreover, the majority of *pex2* and *pex16* mutant flies are unable to fly at 10 days of age (**Fig 3D, S3 Video**- *pex2*^*2*^ flight assay compare to **S4 Video** *pex2*^*2*^ Rescue flight assay; **S5 Video**- *pex16*^*1*^ flight assay compare to **S6 Video** *pex16*^*1*^ Rescue flight assay). To characterize the activity of the *pex2* and *pex16* mutant flies we monitored their activity with the *Drosophila* Activity Monitor (DAM) assay [51]. In this assay activity is constantly monitored by an infrared beam for a continuous 3–7 day period in 12hrs light/dark cycles. We noted that 2-3-day-old *pex2* and *pex16* mutant flies had a dramatic reduction in activity (**Fig 3E**).

In addition, we assessed the function of the *Drosophila* photoreceptors in the *pex* mutant animals with electroretinogram (ERG) recordings (**S2 Fig**). The amplitude of the ERG was measured as the size of change in potential occurring during depolarization, after the synaptic “on” transient and before the “off” transient (**S2A Fig**). There was no significant difference in ERG amplitude in 2-day-old *pex2* nor in 2-day-old *pex16* mutants (**S2A and S2C Fig**, quantification **S2B and S2D**). After 4 weeks of aging the animals in 12 hour light/dark cycle there was a significant reduction in ERG amplitude by approximately one third in *pex2*^*2*^, *pex2*^*1*^ and *pex16*^*1*^ (**S2E and S2G Fig**, quantification **S2F and S2H**). Interestingly, the *pex16*^*EY*^ allele did not exhibit a significant functional change. In summary, *pex2* and *pex16* mutants exhibit similar phenotypes with respect to viability, lifespan, bang sensitivity, flight, photoreceptor function, and locomotor activity.

### *Pex* mutants exhibit dysfunction in carbohydrate metabolism

**Fig 4:**
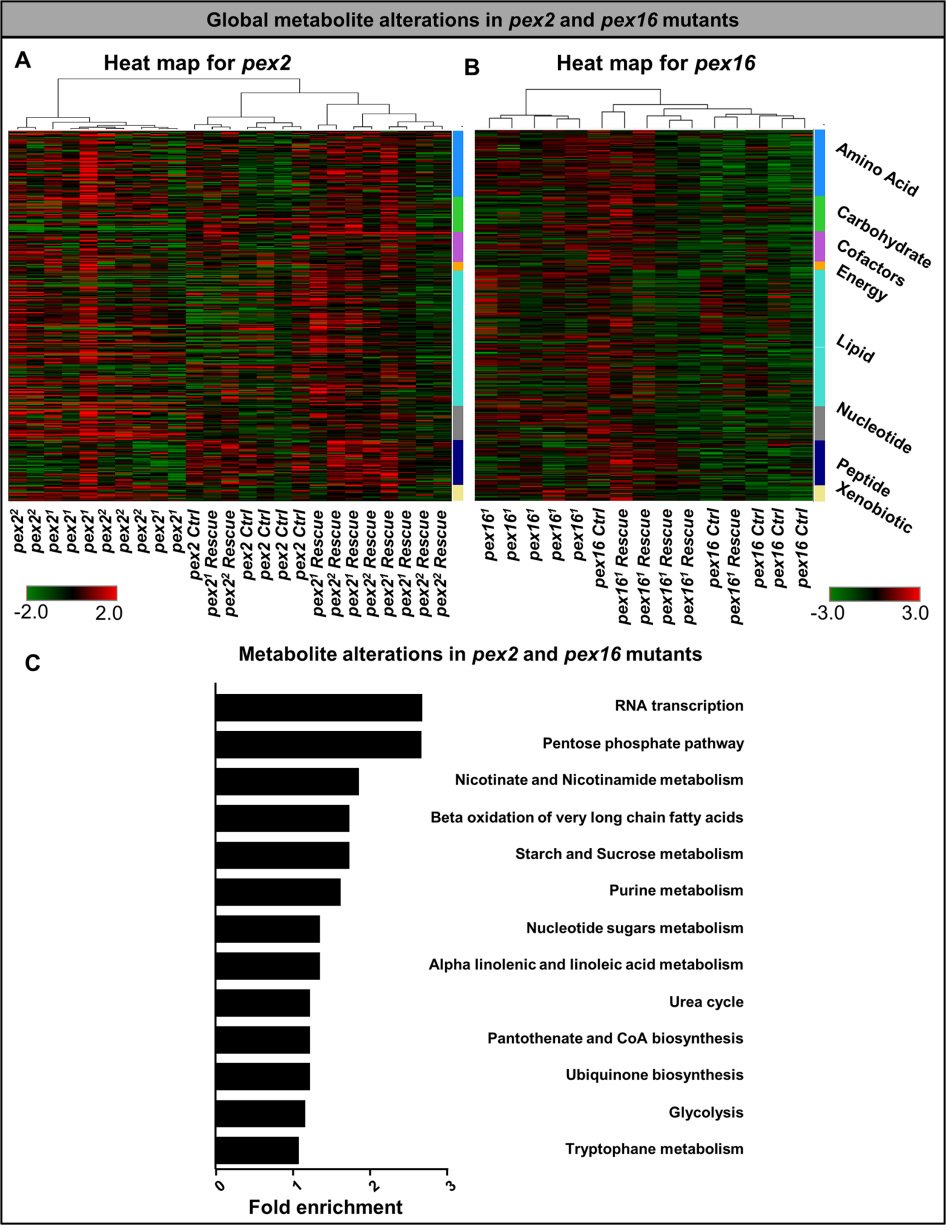
Global metabolomic profiling in *pex2* and *pex16* mutants. (A) Heat map for global metabolomic profiling for the pex2 deletion alleles are shown. Super pathways for amino acids, carbohydrates, cofactors, energy, lipids, nucleotides, peptides and xenobiotics are shown. (B) Heat map for the *pex16* deletion allele. (C) Metabolite set enrichment enrichment was performed on the subset of metabolites that were consistently altered in *pex2* and *pex16* deletion alleles. The fold enrichment values are shown, the fold enrichment is highest for RNA transcription, Pentose-phosphate pathway, Nicotinate and Nicotinamide metabolism, Beta oxidation of VLCFA and starch and sucrose metabolism.

To assess metabolomic changes in two *pex* mutants we undertook a comprehensive metabolomic and characterization of the *pex2* and *pex16* mutants using untargeted metabolomics. We tested the adult mutant flies for 347 named analytes in distinct metabolic pathways. The metabolomic profiles across the 347 compounds is distinct as well as overlapping for *pex2* and *pex16* mutants when compared to control and rescue genotypes (**Fig 4A and 4B, S2 and S3 Tables**). To determine which pathways were enriched for altered metabolites in peroxisomal biogenesis mutants we performed a Metabolite Set Enrichment Analysis (MSEA) [52, 53]. MSEA is analogous to Gene Set Enrichment Analysis (GSEA) in which a set of metabolites is explored for specific biochemical pathways that are enriched for alterations. We selected lists of metabolites that were altered in *pex2* mutants (both alleles) as well as *pex16* null mutants (*pex16*^*1*^ allele) (**S3 Fig**). We also selected the subset of metabolites which were 1) altered consistently between *pex2* (both alleles) and *pex16* (*pex16*^*1*^ allele) compared to rescue (**Fig 4C, S4 Table**). The pathways implicated by MSEA included a broad range of processes. For example, pathways such as RNA transcription were identified owing to abnormal levels of adenosine monophosphate and uridine 5’ monophosphate in the *pex* mutant flies (**Fig 4C, S4 Table**).

**Fig 5:**
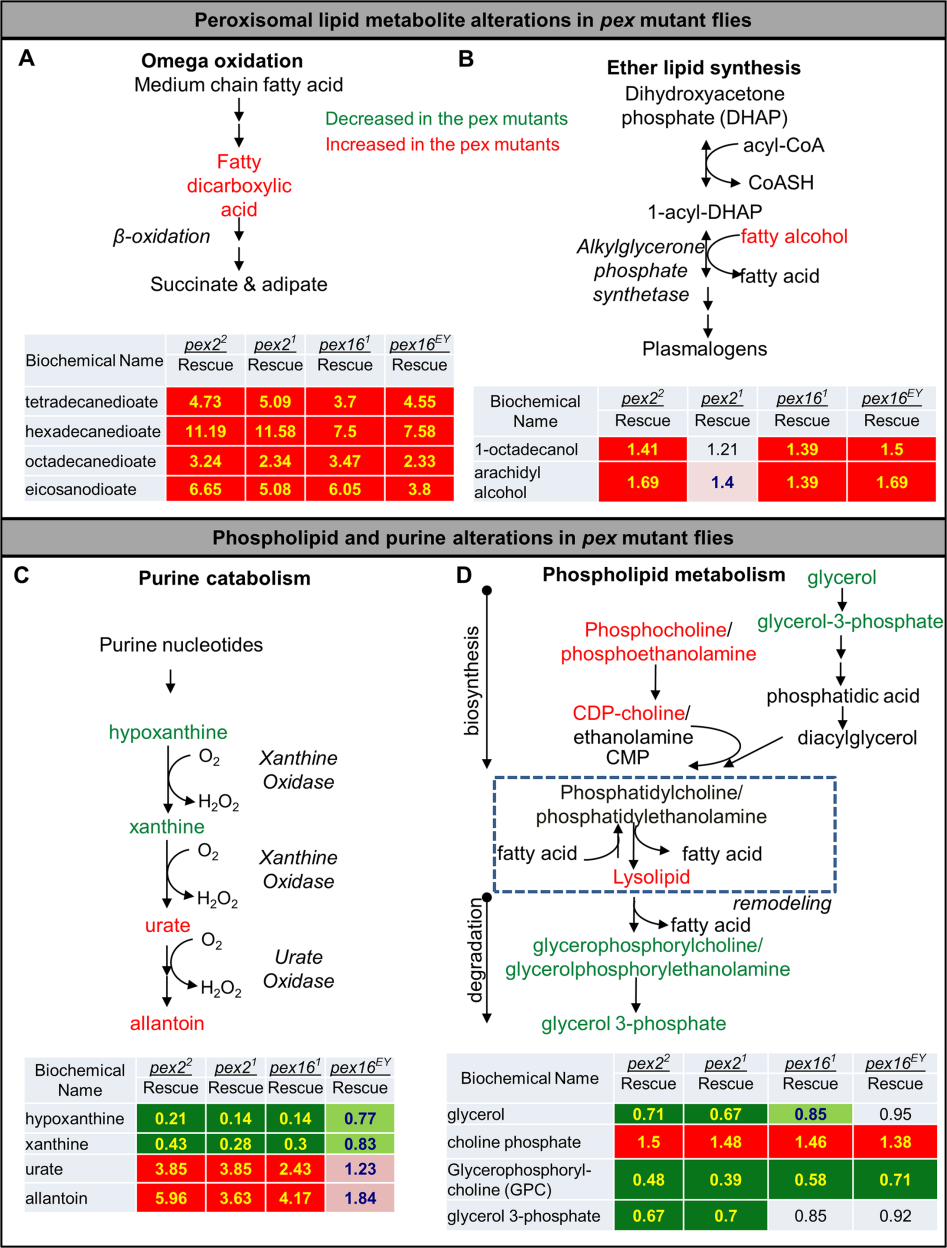
Key pathways with metabolic alterations in *pex* mutant flies. (A) Omega oxidation of medium chain fatty acids can be affected by the Beta-oxidation systems in peroxisomes and we observe numerous fatty dicarboxylic acids accumulating in the *pex* mutant flies. Red indicates analytes in the pathway with increased levels in the mutant versus the genomic rescue line for that mutation. The green indicates decreased levels in the mutant versus the genomic rescue line for that mutant. In the table shown are ratios of analyte levels for each pex allele (*pex2*^*1*^*/Df*, *pex2*^*2*^*/Df*, *pex16*^*1*^*/Df*, *pex16*^*EY*^*/Df*) compared to the corresponding rescue for each allele. Red background with yellow text indicates significant increase while pink background and blue text indicates a value narrowly missing statistical cutoffs. Green background with yellow text indicates significant decrease while light green background with blue text indicates a value narrowly missing statistical cutoffs. Four analytes representing fatty dicarboxylates are shown with significant increases in the pex mutants over the corresponding rescue line for each mutation (see also S1 Table). (B) Ether lipid biosynthesis of plasmalogens relies on two enzymes in peroxisomes and fatty alcohols conjugate with 1-acyl-dihydroxyacetone phosphate. The same color code as (A) is applied, the two fatty alcohol species are shown for the pex mutants over the corresponding rescue line for each mutation. Fatty alcohols appear to accumulate in pex mutant flies. (C) Purine catabolism relies on peroxisomal enzymes and purine metabolites are altered in the pex mutants. The same color code as (A) is applied, hypoxanthine, xanthine, urate and allantoin are shown for the pex mutants over the corresponding rescue line for each mutation. Hypoxanthine and xanthine appear to be reduced while urate and allantoin appear to accumulate in the pex mutants. (D) Phospholipid metabolism is altered in pex mutant flies. The same color code as (A) is applied for the pex mutants over the corresponding rescue line for each mutation.

The examination of biochemical pathways verified several known peroxisomal pathways (**Fig 5**). For example, Omega oxidation of fatty acids can be affected by loss of β-oxidation in peroxisome[54] (**Fig 5A**), and synthesis of ether lipids (plasmalogens) (**Fig 5B**) were perturbed in the *pex2* and *pex16* mutants. Strikingly, dicarboxylic fatty acids of different chain lengths were among the most severely (5–10 fold increased from mutant to rescue lines) and consistently deregulated metabolites (**Fig 5A**). Moreover, defects in purine catabolism were also consistent in the *pex2* and *pex16* mutants [55](**Fig 5C**). Therefore, the untargeted metabolic profiling identified defects in a number of pathways already implicated in peroxisomal biochemistry.

Our data also revealed an increase in precursors for phospholipids such as cytidine 5’-diphosphocholine and phosphoethanolamine, while observing decreases in the degradation products of phospholipids such as glycerophosphorylcholine and glycerophosphorylethanolamine in the *pex2* and *pex16* mutants (**Fig 5D**). This suggests a defect in synthesis and a reduction in the breakdown of membrane lipids such as phosphatidylcholine and phosphatidylethanolamine.

### *Pex* mutants are hypersensitive to lack of sugar

**Fig 6:**
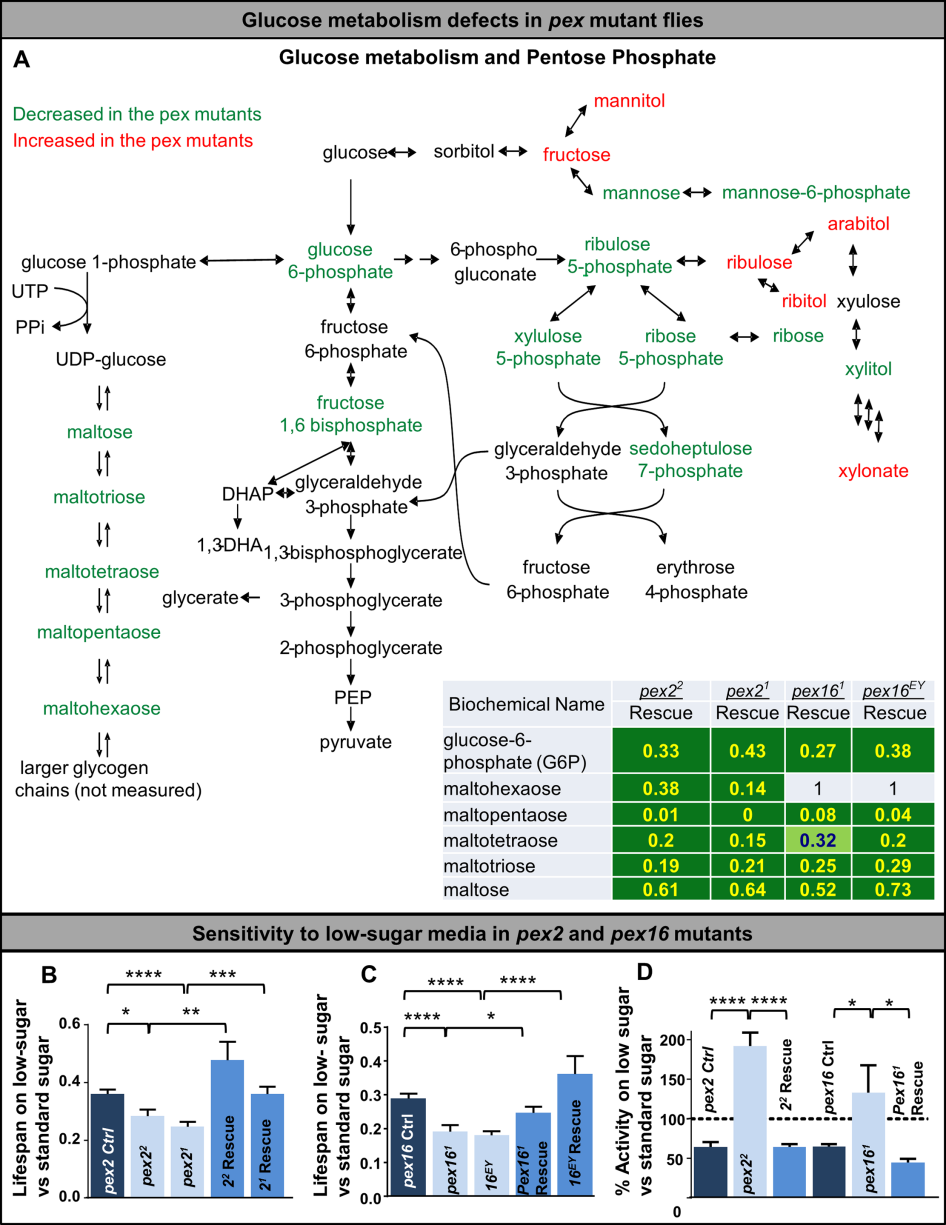
Glucose metabolism defects in *pex* mutant flies. (A) Glucose metabolism alterations in the *pex* mutant flies. Red indicates analytes in the pathway with increased levels in the mutant versus the genomic rescue line for that mutation. The green indicates decreased levels in the mutant versus the genomic rescue line for that mutant. In the table shown are ratios of analyte levels for each pex allele (*pex2*^*1*^*/Df*, *pex2*^*2*^*/Df*, *pex16*^*1*^*/Df*, *pex16*^*EY*^*/Df*) compared to the corresponding rescue for each allele. Green background with yellow text indicates significant decrease while light green background with blue text indicates a trend towards significance, additional analytes in S1 Table. Glycolysis, glycogen synthesis and pentose phosphate pathways exhibit altered metabolites with reduced glucose 6-phosphate and fructose 1,6-bisphosphate. Glycogen intermediates are also reduced in the *pex* mutant flies (with the exception of maltohexaose in the *pex16* mutants). A number of pentose phosphate intermediates such as ribulose 5- phosphate, xylulose 5-phosphate and mannose 6-phosphate are also reduced. (B) Survival analysis in *pex2* mutants grown on low-sugar versus standard sugar. Survival experiments were run at the same time at room temperature on low-sugar or standard sugar (see Materials and Methods). (C) Survival analysis in *pex16* mutants grown on low-sugar versus standard sugar. Survival experiments were run at the same time at room temperature on low-sugar or standard sugar (see Materials and Methods). (D) Total activity was monitored by the DAM assay and the ratio of activity on low-sugar versus regular media was determined for 6 genotypes. While control and rescue genotypes display reduced total activity in low-sugar media, the *pex* mutants increase their activity relative to moderate amounts of sugar. The 100% line refers to average activity on standard sugar media.

Interestingly, the results of MSEA in the group of metabolites consistently altered in both *pex2* and *pex16* mutants pointed to a number of analytes in carbohydrate metabolism (Pentose phosphate pathway, glycolysis, and starch and sucrose metabolism). Indeed a number of compounds in these pathways were altered in the *pex* mutant flies (**Fig 6A**).Since mitochondrial function could underly changes in carbohydrate metabolism and peroxisomes and mitochondria have a number of functional links[56], we examined mitochondria in the *pex* mutant flies (**S4 Fig**). We performed Transmission electron microscopy (TEM) on aged photoreceptors of the hypomorphic *pex16*^*EY*^ flies at 2 weeks (**S4A–S4F Fig**). We observed a statistically significant increase in the number of mitochondria per photoreceptor (**S4B** and **S4E Fig** compared to **S4A** and **S4D**). In addition we observed electron dense inclusions in the photoreceptors of the *pex16*^*EY*^ animals (**S4E Fig** white arrows, **S4F**). These data demonstrate an increase in mitochondrial numbers in *pex16*^*EY*^ photoreceptors. In addition we examined the function of mitochondrial complexes in purified mitochondria from *pex2*^*2*^ compared to *y w*, *and pex2*^*2*^ Rescue (**S4G Fig**). While some minor differences in individual complexes, there were importantly no dramatic reductions in the ETC complexes in the *pex2* mutant flies (**S4G Fig**). While there was a statistically significant reduction in *pex2* mutant flies in complex IV, this was a small, 20% reduction in activity (**S5 Table**). Taken together we did not observe dramatic differences in mitochondrial function in the *pex* mutant flies.

Based on this finding, we examined the carbohydrate metabolism pathways themselves more carefully in our *pex* mutant flies and noted a pattern of reduced glycolytic intermediates such as glucose-6 phosphate (ratio of 0.33–0.43 in pex2 mutants compared to rescue (p<0.05), 0.27–0.38 in pex16 mutants compared to rescue (p<0.05)). We also noted a reduction in glycogen intermediates such as maltose, maltotriose, maltotetraose, maltopentaose and maltohexaose (**Fig 6A**). Given these changes we sought to test whether the peroxisomal mutants would be sensitive to reduced glucose intake. We tested flies for lifespan and locomotor activity on a low-sugar food providing 21 kcal/100 mL with 0% of calories from sugar, compared to a standard sugar food with 55 kcal/100 mL and 88% of calories from sugar. The standard sugar food was most closely related in composition to standard media but is providing nearly 90% of calories from carbohydrate (**S5A and S5B Fig**) (see Materials and Methods).

We noted that the lifespan of both *pex* mutants was dramatically reduced when the flies are raised on low-sugar food (**Fig 6B and 6C, S5C Fig**). We also tested the *pex2* and *pex16* mutants on low-sugar in the DAM assay to score their activity level in these conditions. We observed that control and rescue flies reduce their activity level on low-sugar food. Interestingly, we noted an increase in activity of the mutant flies in the low-sugar food suggesting an altered behavior on low-sugar food (**Fig 6D**). This increased activity was a somewhat surprising response given the evidence for depletion of glycolytic intermediates and glycogen in the pex mutants. Taken together these results suggest a strong physiological carbohydrate dependence in *pex2* and *pex16* mutants *in vivo* which is consistent with the metabolomic analyses.

**Fig 7:**
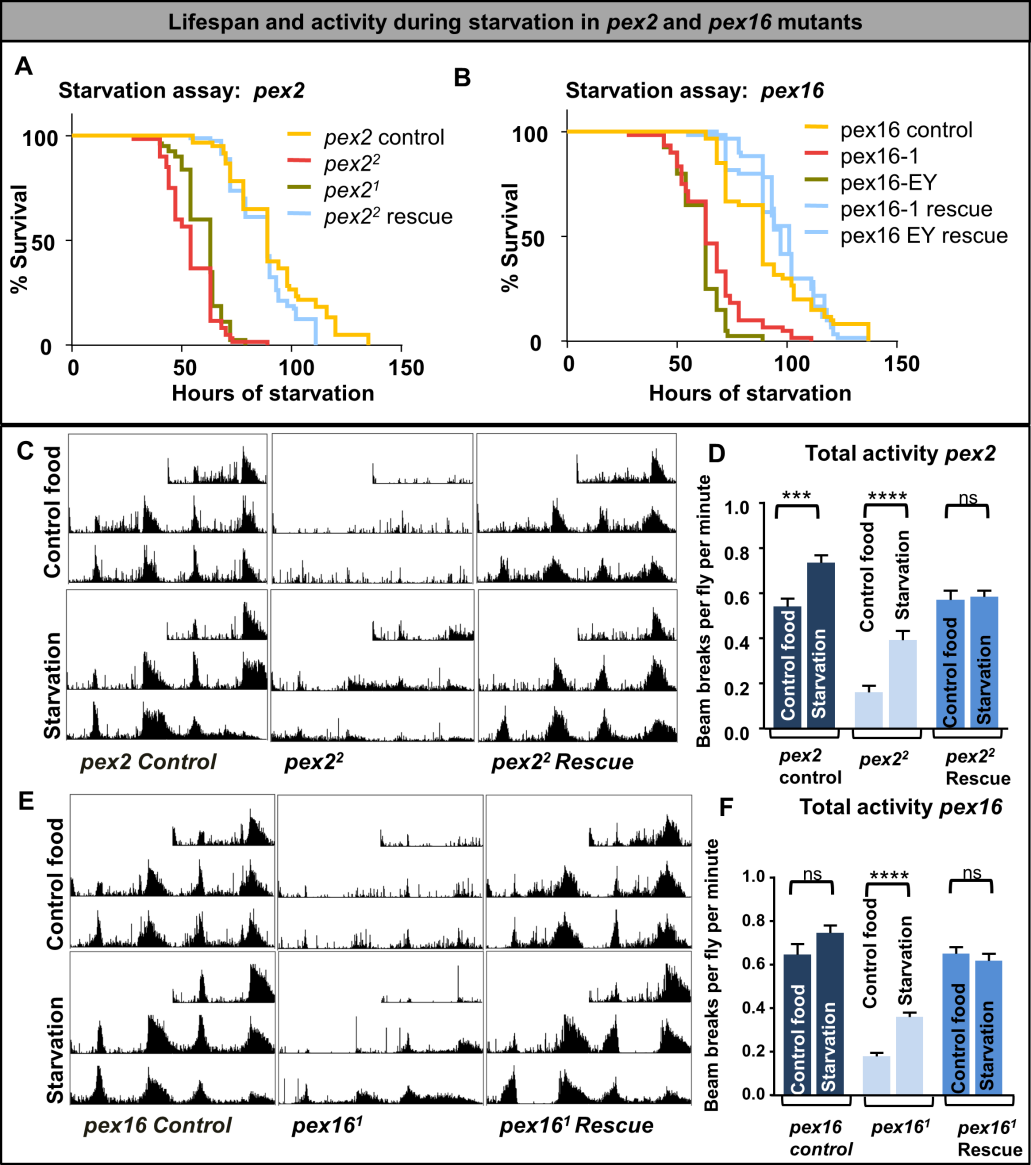
Lifespan and activity during starvation in *pex2* and *pex16* mutants. (A) Survival assay during starvation in *pex2* mutant flies. Color codes show the genotypes with control flies (yellow)(n = 60), *pex2*^*2*^ (red)(n = 60), and *pex2*^*1*^ rescue (blue)(n = 80) and *pex2*^*1*^,(brown)(n = 80). (B) Survival assay during starvation in *pex16* mutant flies. Color codes show the genotypes with control flies (yellow)(n = 60), *pex16*^*1*^ Rescue(n = 60), and pex16^EY^ rescue (solid blue)(n = 60), and *pex16*^*EY*^ (brown)(n = 40) and *pex16*^*1*^,(red) (n = 60). (C) Actograms in the DAM assay in control and starvation conditions showing increased activity level particularly in the *pex2*^*2*^ mutants. (D) Quantification of total activity in the DAM assay in control and starvation conditions (bars indicate SEM). (E) Actograms in the DAM assay in control and starvation conditions showing increased activity level particularly in the *pex16*^*1*^ mutants. (F) Quantification of total activity in the DAM assay in control and starvation conditions (bars indicate SEM).

We also grew adult flies on agar media without nutrients to determine their sensitivity to starvation and measured their activity in the DAM assay during starvation (**Fig 7**). We noted that both *pex2* and *pex16* mutants are sensitive to starvation (**Fig 7A and 7B**). In the DAM assay (**Fig 7C–7F**) the *pex2* and *pex16* mutants, although still severely impaired, display a doubling of their activity under starvation (**Fig 7C and 7E**). These data show an increase in activity during starvation for the *pex* mutants that is more pronounced than in controls. Taken together these data suggest that the *pex* mutants respond differently than controls to changes in carbohydrate supply. In addition, *pex* mutant flies are sensitive to reduced glucose in the media and to starvation, and under both conditions these flies have a an increase in their activity level consistent with foraging behavior.

### Expression profile of peroxisomal biogenesis and carbohydrate metabolism genes are correlated in flies and mice

**Fig 8:**
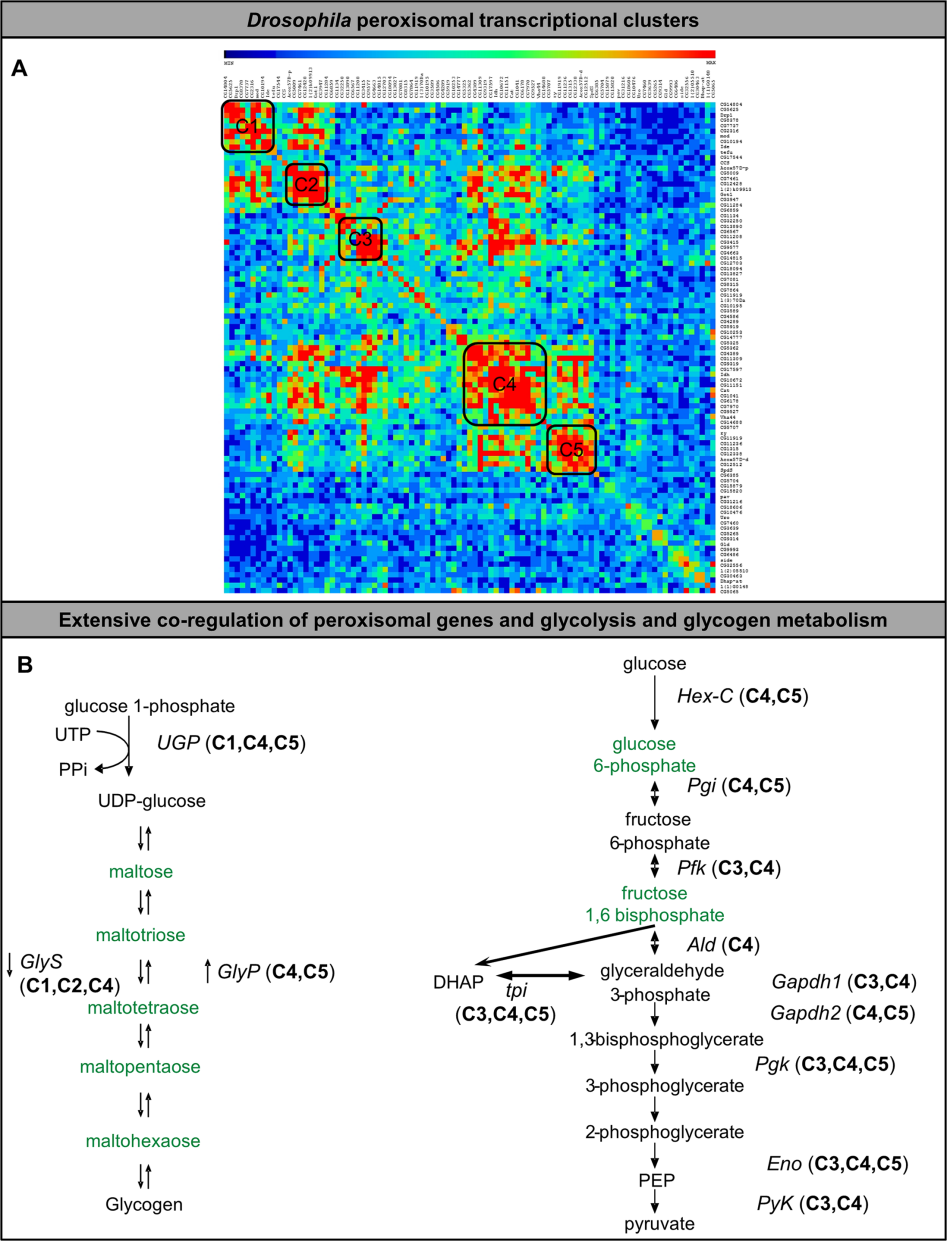
*Drosophila* peroxisomal gene networks. (A) *Drosophila* peroxisomal genes were manually selected and assembled into a correlation matrix using the g:Profiler tool. These *Drosophila* peroxisomal genes are clustered in 5 co-regulated gene clusters that were subsequently used to identify the top 1000 genes for each cluster that has a similar expression pattern from *Drosophila* gene expression data-sets. (B) Co-regulated genes within the top 1000 genes in each cluster include multiple pathways in carbohydrate metabolism including glycogen metaboliwsm and glycolysis The metabolomics results from Fig 6 with green indicating a consistent decrease in metabolite levels in the *pex* mutants, are overlaid with the genes responsible for the relevant steps in metabolism and their representation within the gene cluster (C1 = Cluster 1, C2 = Cluster 2 etc.).

The enzymes of glycolysis, glycogen catabolism and pentose phosphate pathway are not present in peroxisomes [57]. We therefore sought to explore the relationship between genes related to peroxisomal function and carbohydrate metabolism. We first assembled a list of peroxisomal genes in the fly [1, 39, 58] and examined their expression profile in existing databases to find genes whose expression correlates with the expression of these peroxisomal genes using g:Profiler tools [59, 60] (**Fig 8; S6 Table**). This approach revealed five distinct gene clusters containing peroxisomal genes in *Drosophila* (**Fig 8A**). These gene clusters were used to create ranked lists from the *Drosophila* genome of genes whose expression most closely correlated with these gene clusters (**S6 Table**). Next we examined the specific genes encoding enzymes in the glycolysis and glycogen metabolism pathways. Interestingly, for nearly every step of glycogen synthesis and glycolysis the gene encoding the enzyme is co-regulated with one or more of these peroxisomal transcriptional clusters (**Fig 8B**). Taken together these results suggest extensive co-regulation of transcription between peroxisomal genes and carbohydrate metabolism genes in *Drosophila*.

**Fig 9:**
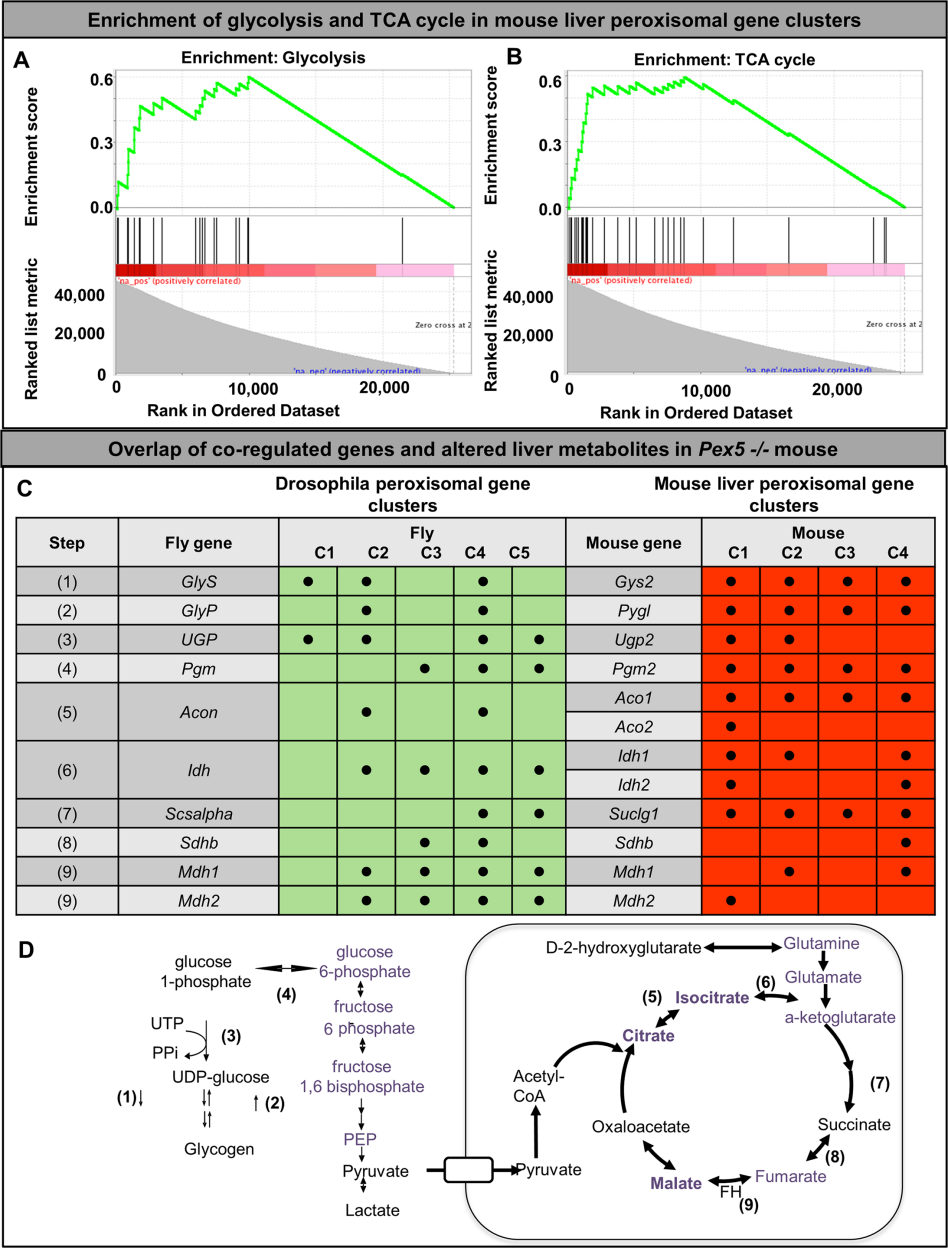
Gene network and metabolomic enrichment of carbohydrate metabolism in peroxisomal gene networks. (A) Gene set enrichment analysis (GSEA) of peroxisomal genes in mouse liver networks showing a strong enrichment for glycolysis. The y-axis shows the enrichment score or Kolmogorov-Smirnov-like statistic representing to what degree genes are over-represented in the ranked list of genes across the murine genome. (B) Gene set enrichment analysis (GSEA) of peroxisomal genes in mouse liver networks showing a strong enrichment for TCA cycle. The y-axis shows the enrichment score or Kolmogorov-Smirnov-like statistic representing to what degree genes are over-represented in the ranked list of genes across the murine genome. (C) Overlap of the *Drosophila* and mouse liver peroxisomal gene clusters. Several genes in carbohydrate metabolism were represented in the top 1000 genes within the *Drosophila* peroxisomal gene cluster and the top 1000 genes within the *Drosophila* murine liver clusters. These genes represent 9 steps in carbohydrate metabolism including glycogen metabolism, and several steps in the TCA cycle. (D) Overlap of the co-regulated steps in carbohydrate metabolism with the altered metabolites seen in Pex5 mouse liver. In purple are altered metabolites in the liver specific Pex5 mouse, and in bold purple are those altered in both the liver specific Pex5 mouse as well as the fetal Pex5 knockout mouse.

Next we sought to explore the conservation of this metabolic control in vertebrates. We undertook an analysis using the g:Profiler starting with a manually curated list of mouse peroxisomal genes which we analyzed informatically in liver transcriptional datasets in mouse (**S5 Fig, S7 Table**) [59]. We observed four distinct clusters of genes that are closely co-regulated. Since we noted a multiple genes implicated in glycolysis, pentose phosphate and glycogen metabolism in the *Drosophila* peroxisomal cluster, we tested whether carbohydrate metabolism genes were enriched in the peroxisomal gene cluster for murine liver and performed a Gene-set enrichment analysis (GSEA) for enzymes in carbohydrate metabolism to test whether there is a statistically significant enrichment in vertebrates for glucose metabolism within the peroxisomal gene clusters. We found a dramatic enrichment for glycolysis (enrichment score 0.60, P<0.0001), and TCA cycle (enrichment score 0.59, P<0.0001) there is a dramatic enrichment (**Fig 9A and 9B**). For genes in gluconeogenesis (enrichment score 0.70 P = 0.022), pentose phosphate pathway (enrichment score 0.67, P = 0.016) and glucose regulation (enrichment score 0.67, P = 0.039) there was a less striking enrichment but still a statistically significant difference. Interestingly, the genes in glycogen metabolism were not significantly enriched (Synthesis, enrichment score 0.72, P = 0.086, Regulation, enrichment score 0.46, P = 0.61) possibly reflecting a smaller number of genes. Therefore, in murine liver there is significant co-regulation of peroxisomal genes and genes involved in carbohydrate metabolism. Based on this pattern of similar co-regulation between peroxisomal genes and carbohydrate metabolism we hypothesized that there may be similarities between the carbohydrate metabolism defect we observed in *Drosophila pex2* and *pex16* mutants and those observed in the *Pex5* liver conditional mouse [36]. *Pex5* conditional liver knockout leads to altered glycolysis, glycogen production and pentose phosphate pathway. In addition, the Pex5 conditional liver knockout exhibits activation of AMP-activated protein kinase (AMPK) pathway and suppression of PPAR-γ and PGC-1α [36]. Of note, *Drosophila* do not have a PPAR-γ homolog but indeed we observe that the *Drosophila* gene clusters included several target genes for AMPK and PGC-1α with many AMPK targets appearing in Cluster 1 (**S8 Table**)[61, 62]. In addition, amongst the top 1000 genes from each gene cluster we selected genes that are 1) involved in glucose metabolism and 2) represented in the top 1000 of more than one peroxisomal gene cluster in both fly and mouse. This selection identified 10 fly genes, corresponding to 12 mouse homologs in glucose metabolism that are strongly co-regulated with peroxisomal genes in both flies and mice. These genes catalyze 10 steps in glucose metabolism (**Fig 9C and 9D**), 4 steps in glycogen metabolism and six steps in the TCA cycle.

### *Pex5* mice livers exhibit altered carbohydrate metabolism

We hypothesized that this co-regulation in these key steps of carbohydrate metabolism might relate to the peroxisome’s dependence on other pathways such as the citric acid cycle to metabolize the end products of peroxisomal catabolism[63]. This would lead to the prediction of altered citric acid cycle metabolites in peroxisomal biogenesis mutants. We therefore undertook additional metabolomic studies in *Pex5* mice [32, 36]. Adult mouse liver was examined in the liver conditional *Pex5* mice (L-*Pex5* mice) [36]. The global *Pex5* knockout mice were also examined but we could only test fetal liver because these mice usually die at P1 [32]. We used LC-MS to assess a targeted panel of glucose and TCA cycle metabolites in fetal and adult *Pex5* liver tissue and found that for both fetal and adult *Pex5* liver, the targeted platform could distinguish the mutants as exhibiting a distinct signature of TCA cycle metabolites (**S7 Fig)** Interestingly, we noted a clear correspondence between the significantly altered metabolites in the adult conditional *Pex5* liver and the strongly co-regulated steps in glucose metabolism (**Fig 9D**, purple analytes). For the fetal liver the only significantly altered metabolites, malate and citrate/isocitrate also corresponded to strongly co-regulated steps (**S7 Fig)**. Taken together these data indicated consistency between the transcriptional evidence for co-regulation of peroxisomes with the TCA cycle and metabolic abnormalities in PBD models. The genes are all enriched in multiple peroxisomal gene clusters in fly and mouse. In conclusion, the most strongly co-regulated steps of carbohydate metabolism with peroxisomal genes between fly and mouse correspond to metabolomic changes seen in liver from the *Pex5*knockout mice.

## Discussion

Peroxisomes are subcellular organelles tasked with a discrete subset of metabolic reactions principally involving peroxisomal lipids. While a role for lipid metabolism in peroxisomal disorders is well established, carbohydrate metabolism is thought to be a more central energy-producing process utilizing cytosolic and mitochondrial enzymes crucial for energy production and is not generally implicated in PBD. We have uncovered a previously unappreciated metabolic, phenotypic and gene-expression link between peroxisomes and carbohydrate metabolism. We identified co-regulation of peroxisomal genes and carbohydrate metabolic genes along with a carbohydrate dependence phenotype in peroxisomal biogenesis mutants with metabolomic studies in *Drosophila*. In addition, we link this defect to that observed in mouse liver tissue suggesting these pathways are conserved.

Our studies in *Drosophila* represent a new approach to the studies of *Drosophila pex* mutants. We studied *pex2* and *pex16* mutant flies in order to compare different biogenesis defects in the fly. Previous studies did not report similar phenotypes in *pex2* and *pex16* mutants which was surprising given their strong conservation and similarities in yeast and vertebrates [41, 42]. However, our study rigorously compared different genetic backgrounds by utilizing two alleles for each gene, studying mutants in trans with genomic deficiencies and creating genomic rescue strains for each mutation. We determined that peroxisomes are similarly functionally and morphologically defective in both *pex2* and *pex16* mutants. Our functional analysis of the peroxisomal lipids allowed a more comprehensive study of VLCFA metabolites in different stages of development. We also analyzed plasmalogens which had not been characterized previously in *Drosophila*. We observed a dramatic loss of PlsEtn 18:1/20:4 plasmalogen in *pex2* and *pex16* mutant flies which confirmed a conserved role for the peroxisome in plasmalogen biosynthesis pathway. We note that *pex2* and *pex16* mutants display very similar phenotypes including short lifespan, increased bang sensitivity, lack of flight and reduced activity. The consistency of our results could stem from our use of rigorous controls for genetic background, and allowed us to provide downstream metabolomic studies.

Consistent with a shared peroxisomal biogenesis phenotype in *Drosophila*, metabolomic studies of *pex2* and *pex16* mutants revealed numerous shared metabolic abnormalities that are due to global peroxisomal dysfunction in *Drosophila*. Many of these were expected to result from peroxisomal dysfunction and had been seen in other organisms from previous targeted methods including fatty alcohols and purine metabolites[1]. However, our metabolomics analysis also revealed new insights indicating that the use of untargeted metabolomics in peroxisomal studies could uncover unsuspected metabolic pathways involved in peroxisomal biochemistry. Specifically we observed accumulations of phospholipid precursors and reduction in phospholipid degradation products. More importantly, we found that *Drosophila* peroxisomal biogenesis mutants have global reductions in glycolytic intermediates and glycogen with abnormalities in the pentose phosphate pathway. These unanticipated data allowed us to hypothesize that *pex2* and *pex16* mutants are starvation sensitive and sensitive to glucose deprivation in their diet which we experimentally verified. Of note they exhibit an increase in basal activity under both starvation and low glucose diet, while control flies exhibit starvation-related hyperactivity but seem to reduce their activity in low-sugar. These data demonstrate a particular sensitivity of peroxisomal mutants to glucose deprivation, suggesting that the metabolomics changes we observed in carbohydrate metabolism impact the physiology and behavior of peroxisomal mutant flies. This pathological hyperactivity is to some extent consistent with a starvation hyperactivity that has been observed in flies with altered adipokinetic hormones and octopamine levels [64, 65].

There are several possible explanations for how peroxisomal biogenesis mutations can perturb carbohydrate metabolism. One is that some enzymatic components of glycolysis or glucose metabolism are in the peroxisome. Indeed, Trypanosomes have the entire glycolytic machinery within a peroxisome-like organelle called the glycosome[66]. While this is not supported by the current inventory of peroxisomal proteins in *Drosophila* [39], mechanisms such as read-through at stop codons can lead to peroxisomal localization of some metabolic enzymes in yeast[67]. Another possibility is a secondary effect on metabolism, such as alteration in mitochondria. While we did not observe dramatic mitochondrial phenotypes in the mutant flies (**S5 Fig**), we cannot rule out this possibility. In addition, we observe many metabolic abnormalities in our flies that are observed in *Pex5* conditional liver knock-out mice [36]. *Pex5* conditional liver knockout mice exhibit altered glycolysis, glycogen production and pentose phosphate pathway and these livers exhibit altered glucose regulation with AMP-activated protein kinase (AMPK) activation and PGC-1α suppression [36]. Indeed, we observed that target genes in the AMPK and PGC-1α pathway are represented in the gene clusters we identified in flies (**S8 Table**). While we did observe more dramatic changes to the TCA cycle in the mice, and more dramatic changes in glycolysis in the flies, we see that in both fly and mouse liver peroxisomal biogenesis and carbohydrate metabolism interplay in the same transcriptional gene networks. These also allow us to identify additional pathways as the significant co-regulation may suggest that additional pathways beyond AMPK and PGC-1α may be involved in coordinating peroxisomes and carbohydrate metabolism. Recent studies have identified that the activation of the mTORC1 pathway in response to ROS occurs on the peroxisomal membrane, a process dependent on ATM [68, 69]. Interestingly, some mTOR target genes such as *eIF-4E* and *Cbp80* were also strongly co-regulated with peroxisomes in *Drosophila* (**S6 Table**). We find that both flies and mice strongly co-regulate peroxisomal genes and carbohydrate metabolizing genes, and noticed that twelve vertebrate genes are co-regulated with the corresponding ten fly genes. Similarly, the metabolites corresponding to those steps in carbohydrate metabolism are altered in both species when *pex*/*Pex* genes are mutated. These data suggest evolutionary conservation of the link between carbohydrate metabolism and peroxisomal biogenesis genes.

Our work adds to a growing realization that peroxisomal function is a process that interplays with other metabolic pathways, and suggests that PBD pathogenesis may extend into other metabolic pathways beyond peroxisomal enzymes [29, 70]. Evolutionarily, ancestral peroxisomes were crucial in allowing ancient eukaryotes to detoxify the byproducts of oxygen and performing β-oxidation [56]. In higher eukaryotes β-oxidation occurs in the mitochondria with the exception of peroxisomal oxidation of VLCFA and some other carboxylates. Peroxisomes in these higher eukaryotes are responsible for steps in oxidative metabolism of lipids but they ultimately depend on the TCA cycle to fully metabolize the lipids [63]. Thus, the co-regulation of genes involved in carbohydrate and fat metabolism may have evolved through this interdependence of peroxisomes and mitochondria and maybe the basis of the strong inter-relationship we observed between peroxisomes and carbohydrate metabolism. Hence, examination of the latter pathways in humans may provide additional mechanistic insights and therapeutic targets for PBD. This dataset will provide valuable entry points for those studies.

We have utilized *Drosophila* to identify a key role for glucose metabolism in *pex2* and *pex16* mutants. We find that the same key phenotypes observed in *pex2* and *pex16*, namely their longevity and their locomotor activity, were modifiable by diet and specifically were exquisitely sensitive to starvation stress and reduced glucose media. Interestingly, the *Drosophila* mutants are somewhat comparable to the findings in *Pex5* conditional liver knockout mice which also have reduced glycolytic and glycogen intermediates, and diminished body weight despite increased food intake and carbohydrate dependence [36].

Finally, our work points to the importance of peroxisomal gene regulation in understanding not only peroxisomal biology but also in understanding PBD. We observed closely co-regulated groups of genes in flies and mice. These gene networks were seeded with bona fide peroxisomal genes, but the data suggested close co-regulation between these peroxisomal genes and enzymes in other pathways of metabolism in other cellular organelles. This suggests that the fundamental link in metabolism between peroxisomes and other organelles is regulated. Because peroxisomes lack a TCA cycle but they ultimately rely on mitochondrial TCA cycle for complete oxidation of their metabolites, it seems likely that common transcriptional programs activate peroxisomes and TCA cycle components. Our work suggests that delineating these gene regulatory programs, and how they are altered in PBD is important to our understanding of how defective peroxisomal biogenesis impacts human health.

## Materials and methods

### Fly strains and maintenance

All flies were maintained at room temperature (21°C) and except where otherwise noted experiments were conducted at room temperature. The *pex2*^*1*^, and *pex2*^*2*^ lines were derived from imprecise excision of (*w*[1118]; P{*w*[+mC] = EPg}*pex2*[HP35039]/TM3, Sb[1], see below) these were then backcrossed 5 generations with *y w*: FRT80B and studied as:

y w; FRT80B
y w; FRT80B- pex2^1^
y w; FRT80B- pex2^2^
w[1118];PBac{y[+mDint2] w[+mC] = 53M21}VK00037; FRT80B- pex2^2^
w[1118];PBac{y[+mDint2] w[+mC] = 53M21}VK00037; FRT80B- pex2^1^

Except where otherwise indicated the 5 strains above each crossed to a genomic deficiency uncovering *pex2* locus *w^1118^; Df(3L)BSC376/TM6C, Sb^1^ cu^1^* are labeled as *pex2 Ctrl*, *pex2*^*2*^ *pex2*^*1*^ *pex2*^*2*^ Rescue *and pex2*^*1*^ Rescue respectively.

The *y w*: *pe16*^*1*^ line [41] was obtained from Kenji Matsuno, derived from: *y*^*1*^*w*^*67c23*^; P{GSV6}*pex16*^GS14106^/TM3, Sb^1^ Ser^1^.

The y w: *pex16*^*EY*^ strain was obtained from Bloominton Stock center *y[1] w[67c23]; P{w[+mC] y[+mDint2] = EPgy2}Pex16[EY05323]*.

y w; FRT80B
y w; pex16^1^
y[1] w[67c23]; P{w[+mC] y[+mDint2] = EPgy2}pex16[EY05323]
w[1118];PBac{y[+mDint2] w[+mC] = 115M13}VK00037; pex16^1^
w[1118];PBac{y[+mDint2] w[+mC] = 115M13}VK00037; P{w[+mC]

Except where otherwise indicated the 5 strains above each crossed to a genomic deficiency uncovering the pex16 locus *w^1118^; Df(3L)BSC563/TM6C, cu^1^ Sb^1^* are labeled as *pex16 Ctrl*, *pex16*^*1*^ *pex16*^*EY*^ *pex16*^*1*^ Rescue *and pex16*^*EY*^ Rescue respectively.

### *pex2* excisions

*w[1118]; P{w[+mC] = EPg}pex2[HP35039]/TM3, Sb[1]* was crossed to *y w; CyO*, *delta2-3/Egf*r and progeny crossed to *y w; D/TM6B*, *Tb*. Progeny were screened for loss of *w+* marker. 461 independent excision lines were screened by PCR. *pex2-1* a 7bp insertion within a 606 bp deletion; and *pex2-2* a 11bp insertion within a 473 bp deletion were selected.

### Peroxisomal marker strains

y[1] w[*]; P{w[+mC] = Act5C-GAL4}25FO1/CyO, y[+] second chromosome Actin GAL4 was recombined with a 2^nd^ chromosome UAS-GFP-SKL transgenic (courtesy of Hamed Jefar-Nejad). Recombinants were scored by GFP expression and balanced over *CyO*. These strains were crossed into deficiency strains for *pex2* and *pex16* marker experiments.

### P[acman] rescue strains

For *pex2* a genomic clone, CH322-53M21 from the CHORI-322_BAC collection was obtained. For *pex16* a genomic clone, CH322-115M13 from the CHORI-322_BAC was obtained. These plasmids were amplified and grown, then purified and injected into *y[1] w[1118]; PBac{y[+]-attP}VK00037* embryos (VK00037 contains an attP on 2L at 22A3). Transformants were selected based on the w+ marker and these strains were balanced to generate:

y[1] w[*]; Dp(3;2)CH322-53M21, PBac{y[+mDint2] w[+mC] = CH322-53M21}VK00037
y[1] w[*]; Dp(3;2)CH322-115M13, PBac{y[+mDint2] w[+mC] = CH322-115M13}VK00037

These lines were crossed into the mutant strains listed above.

### *Drosophila* peroxisomal lipid studies

Very long chain fatty acid levels were measured as described by GC/MS [49, 71]. lysophosphatidyl choline (26:0 LPC, and 24:0 LPC) and individual ethanolamine and plasmalogen species by LC-MSMS [47, 48, 50].

### Dissection and immunostaining

For salivary gland staining, dissection of third instar larvae was performed and larvae were fixed in 3.7% formaldehyde for 20 min at room temperature and washed in PBS containing 0.4% Triton X-100. The primary antibody were used at the following dilution: chicken anti-GFP 1:1000 (AB13970, Abcam, Cambridge, MA), rabbit anti-pex3 1:500 (From McNew lab, Rice U). Donkey anti Chicken Alexa 488 conjugated (#703-545-155, Jackson ImmunoResearch, PA) and Goat anti-rabbit Cy3 conjugated secondary antibodies (#111-165-003, Jackson ImmunoResearch, PA) were used at 1:250. DAPI (D1306, ThermoFisher) was used at 300nM. Samples were mounted in Vectashield (Vector Labs, Burlingame, CA).

### Lifespan determination

Flies were collected under CO_2_ between 1 and 24 hours after eclosion. Male and female flies were separated and flies were kept 10 flies per vial at room temperature and the fly food was changed every 3 days. A tally of number of live flies was kept, and the number of live flies was checked every 3 days until the last fly had died. Data was analyzed with a Kaplan-Meier survival curve.

### Bang sensitivity

Flies were kept without exposure to C0_2_ for at least 48 hours prior to the assay. Flies were vortexed for 10 seconds in a vial, and a video recording was made of each trial. Video recordings were analyzed separately and blinded to genotype for recovery time for each fly.

### Flight assay

Flies were kept without exposure to C0_2_ for at least 48 hours prior to the assay. Flight assay was performed in 10 day old flies. Flies of the 10 indicated genotypes were lightly tapped into a clear column made of PVC marked at each centimeter. Video recordings of each trial were made and analyzed separately and blinded to genotype for the landing height of each fly.

### *Drosophila* activity monitoring assay

A *Drosophila* activity monitoring system was used to study activity as described[51]. Briefly, adult flies were placed in small tubes with food, and kept at 25°C in a 12 hour light/dark cycle for 24–48 hours before being placed in the monitor. The monitor was temperature-controlled at 25°C in a 12 hour light dark cycle. Flies were kept in the monitor for 3–7 days for the various experiments described.

### Electroretinograms

Flies of the indicated genotypes were aged for either 2 days or 4 weeks after eclosure in a 12 hour light dark cycle. Electroretinograms were performed as described[72]. Briefly, adult flies were glued to glass slides. A recording electrode was placed onto the surface of the eye while another reference electrode was inserted into the cuticle in the posterior portion of the head. The eyes were then exposed to controlled sudden flashes of white light and the response was recorded and analyzed using AXON-pCLAMP8.

### Transmission electron microscopy

TEM of photoreceptor terminal were performed on 2 week aged flies as described[73]. Briefly, fly heads or third instar larva were dissected and fixed at 4°C in 4% paraformaldehyde, 2% glutaraldehyde, 0.1 M sodium cacodylate, and 0.005% CaCl_2_ (PH 7.2) overnight, post-fixed in 1% OsO_4_, dehydrated in ethanol and propylene oxide, and then embedded in Embed-812 resin (Electron Microscopy Sciences, Hatfield, PA). Photoreceptors were then sectioned and stained in 4% uranyl acetate and 2.5% lead nitrate. TEM images of PR sections were taken using a JEOL JEM 1010 transmission electron microscope with an AMT XR-16 mid-mount 16 mega-pixel digital camera.

### Mitochondrial electron transport chain

Mitochondrial Electron transport chain activity was measured on isolated mitochondria extracted as previously described[74, 75] Each ETC complex activity was quantified as nmoles/min/mg protein, normalized to citrate synthase activity, and expressed as %control activity.” (**S5 Table**).

### Untargeted metabolomics

We used an untargeted metabolomics platform through Metabolon. This platform uses ultrahigh performance liquid chromatography/electrospray ionization tandem mass spectrometery [76]. The raw analyte values in this platform are analyzed by performing a z-score calculation compared to mean and standard deviation in other clinical samples. Welch’s two sample t-tests are used to determine significant alterations and multiple comparisons are accounted for with the false discovery rate method.

### Food conditions

In all experiments except where otherwise indicated, flies were grown on a standard media comprised of Agar, molasses, corn meal, dried yeast, proprionic acid, methyl p-hydroxybenzoate. Food for starvation was comprised of 2% agar only. The conditional food conditions (Figs 6 and 7) consist of agar, cornmeal, yeast (Sigma), dextrose (Sigma), sucrose (Sigma), methyl para-hydroxybenzoate and propionic acid (Fisher Scientific). Recipe for every 100ml has 0.6 grams agar, 0.5ml methyl para-hydroxybenzoate, and 0.75ml propionic acid in common. For other ingredients, low-sugar food has 7.5 grams yeast; standard sugar food has 6 grams cornmeal, 1.5 grams yeast, 5 grams dextrose, and 2.5 grams sucrose. Ingredients including agar, cornmeal, yeast, dextrose, and sucrose were added to a cooking vessel, mixed then brought to a boil then immediately covered, mixed and cooled under 70°C at which time methyl para-hydroxybenzoate and propionic acid were added. The food was then poured into vials for experiments.

### Gene network analysis

*Drosophila* and murine liver gene network analysis was performed using g:Profiler essentially as described [59, 60].

### Targeted TCA metabolites in mouse liver with mass spectrometry

Mouse liver samples were provided by Myrian Baes. All animal work was conducted according to the guidelines for humane treatment of animals.

#### Reagents and internal standards

High-performance liquid chromatography (HPLC) grade acetonitrile, methanol and water were purchased from Burdick & Jackson (Morristown, NJ). Mass spectrometry grade formic acid and internal standards namely, Tryptophan-15N2, Glutamic acid-d5, Thymine-d4, Gibberellic acid, Trans-Zeatin, Jasmonic acid, Anthranilic acid, and Testosterone-d3 were purchased from Sigma- Aldrich (St.Louis, MO). The calibration solution containing multiple calibrants in acetonitrile/trifluroacetic acid /water was purchased from Agilent Technologies (Santa Clara, CA).

#### Sample preparation for mass spectrometric analysis

The metabolome extraction method described earlier was used for the liver tissue in this study. Briefly, cells were thawed at 4 ^o^C and subjected to freeze-thaw cycle in liquid nitrogen and over the ice three times to rupture the cell membrane. Following this, an 750 μL of ice cold methanol: water (4:1) containing 20 μL of spiked internal standards (8) was added to each tissue sample. The cells were homogenized for 1 min (30 sec pulse twice) and mixed with 450 μl of ice cold chloroform and vortex mixed in a Multi-Tube Vortexer for 10 min. The resulting homogenate was mixed with 150 μl of ice cold water and vortexed again for 2 min. The homogenate was incubated at -20 ^o^C for 20 min and centrifuged at 4^°^C for 10 min to partition the aqueous and organic layers. The aqueous and organic layers were separated and dried at 37^°^C for 45 min in an Automatic Environmental Speed Vac® system (Thermo Fisher Scientific, Rockford, IL). The aqueous extract was reconstituted in 500 μl of ice cold methanol:water (50:50) and filtered through 3 kDa molecular filter (Amicon Ultracel -3K Membrane, Millipore Corporation, Billerica, MA) at 4 ^o^C for 90 min to remove proteins. The filtrate was dried at 37^o^ C for 45 min in speed vac and stored at -80^°^C until mass spectrometry analysis. Prior to mass spectrometry analysis, the dried extract was resuspended in 100 μL of methanol:water (50:50) containing 0.1% formic acid and analyzed using MRM.

The tissues were stored at −140°C in liquid nitrogen until analysis. For extraction of the metabolome, 25 mg of tissue was homogenized in 1:4 ice-cold water:methanol mixture containing an equimolar mixture of 8 internal standard compounds. This was followed by metabolic extraction using sequential application of ice-cold organic and aqueous solvents (water:methanol:chloroform:water; ratio 1:4:3:1), deproteinization and drying of the extract as mentioned above.

Liquid Chromatography- Mass spectrometry HPLC analysis was performed using an Agilent 1290 series HPLC system equipped with a degasser, binary pump, thermostatted autosampler and column oven (all from Agilent Technologies, Santa Clara, CA). The Multiple Reaction Monitoring (MRM)-based measurement of relative metabolite levels, used either reverse phase or normal phase chromatographic separation. All samples were kept at 4 C and 5 μl was used for analysis.

#### Separation of TCA metabolites

Targeting the metabolites, the normal phase chromatographic separation was also used for targeted identification of metabolites. This employed solvents containing water (solvent A), with solvent A modified by the addition of 5mM Ammonium acetate (pH 9.9), and 100% acetonitrile (ACN) solvent B). The binary pump flow rate was 0.2 ml/min with a gradient spanning 80% B to 2% B over a 20 minute period followed by 2% B to 80% B for a 5 min period and followed by 80% B for 13 minute time period. The flow rate was gradually increased during the separation from 0.2 mL/min (0–20 mins), 0.3 mL/min (20.1–25 min), 0.35 mL/min (25–30 min), 0.4 mL/min (30–37.99 min) and finally set at 0.2 mL/min (5 min). Metabolites were separated on a Luna Amino (NH_2_) column (4um, 100A 2.1x150mm, Phenominex), that was maintained in a temperature controlled chamber (37^°^C). All the columns used in this study were washed and reconditioned after every 50 injections.

## Supporting information

S1 Fig Genomic rescue strains for peroxisomal biogenesis factors *pex2* and *pex16*. (A) The *pex2* gene is shown in genomic context, the blue bar represents the specific genomic rescue construct which was used to produce a transgenic line for rescue experiments. A red box indicates the exons shown in Fig 1A.(B) The pex16 gene is shown in genomic context, the blue bar represents the specific genomic rescue construct which was used to produce a transgenic line for rescue experiments. A red box indicates the exons shown in Fig 1B.
(TIF)

S2 Fig Electroretinograms in young and aged *pex2* and *pex16* mutants. (A) Electroretingrams demonstrate the field potential after light exposure in photoreceptors, the amplitude of depolarization (red bracket) was assessed after the “on” and before the “off” transient indicating synaptic activity. The pex2 tracings show no differences between mutant and rescue animal for the indicated genotypes(B) Quantification of the amplitude for 2-day *pex2* flies student’s t-test, P>0.05 = ns, P< 0.05 = *, P<0.01 = **, P<0.001 = ***.(C) The pex16 tracings show no differences between mutant and rescue animal for the indicated genotypes(D) Quantification of the amplitude for 2-day *pex16* flies student’s t-test, P>0.05 = ns, P< 0.05 = *, P<0.01 = **, P<0.001 = ***.(E) The pex2 tracings show reduced amplitude in the mutants between mutant and rescue animals at 4 weeks after 12 hour light-dark cycle.(F) Quantification of the amplitude for 4-week *pex2* flies showing a statistically significant reduction in amplitude in the mutants.(G) The pex16 tracings show reduced amplitude in the mutants between mutant and rescue animals at 4 weeks for the *pex161* allele but not for the *pex16EY*.(H) Quantification of the amplitude for 4-week *pex16-1* flies showing a statistically significant reduction in amplitude in the mutants.
(TIF)

S3 Fig Altered metabolic pathways in *pex2* and *pex16* mutants. (A) Metabolite set enrichment fold enrichment was performed on the subset of metabolites that were consistently altered in *pex2* both deletion alleles. The fold enrichment values are shown.(B) Metabolite set enrichment fold enrichment was performed on the subset of metabolites that were consistently altered in *pex16* deletion allele. The fold enrichment values are shown.
(TIF)

S4 Fig Mitochondrial phenotypes in the *pex* mutant flies. (A) Transmission electron microscopy (TEM) of *Drosophila* photoreceptors. Normal ultrastructure of the photoreceptors in the retina in 2 week old *pex16*^*EY*^ Rescue animals with seven photoreceptors, the dark rhabdomeres and the mitochondria which often cluster in the cell body of the photoreceptor.(B) TEM of *pex16*^*EY*^ animals showing apparent increase in the number of mitochondria per photoreceptor terminal.(C) Quantification of mitochondria per photoreceptor.(D) Inset of A showing mitochondria in the photoreceptor(E) Inset of B showing mitochondria and electron dense inclusions.(F) Quantification of E.(G) Mitochondrial electron transport chain activity in the pex2 mutants. Stars indicate activities with statistically significant differences from the control activity.
(TIF)

S5 Fig Altered mouse liver TCA metabolites in targeted metabolomics. (A) Composition of the food for the conditional food experiments. Total calories per 100 mL of food is shown.(B) Percent of calories for conditional food.(C) Kaplan-Meier curves for the quantification shown in **Fig 8B** and **8C**.
(TIF)

S6 Fig Mouse liver peroxisomal gene network clusters. Mouse liver peroxisomal gene clusters, mouse liver peroxisomal genes are grouped into 4 closely co-regulated clusters.
(TIF)

S7 Fig Altered mouse liver TCA metabolites in targeted metabolomics. (A) Heat map of Pex5 knockout mice versus controls showing some alterations in citrate and malate(B) Heat map of Pex5 liver conditional mice versus controls showing a number of altered analytes including G6P/F6P, citrate, ketoglutarate, glutamate, fumarate and malate.(C) Abundance of Citrate/Isocitrate in Targeted metabolomics in adult and fetal mouse liver showing increased abundance in both global and conditional *Pex5* murine liver compared to controls.(D) Abundance of Malate in Targeted metabolomics in adult and fetal mouse liver showing increased abundance in both global and conditional *Pex5* murine liver compared to controls.
(TIF)

S1 Video Bang sensitivity of *pex2* mutant flies. Bang sensitivity assay in pex mutant flies. Flies were assayed in graduated vials with *pex2* control at left, *pex2*^*2*^ at center and *pex2*^*2*^ Rescue at right. All flies were at 3 days of age were subjected in vials to vortex for 10 seconds immediately prior to the video shown.
(MOV)

S2 Video Bang sensitivity of *pex16* mutant flies. Bang sensitivity assay in pex mutant flies. Flies were assayed in graduated vials with *pex2* control at left, *pex2*^*2*^ at center and *pex2*^*2*^ Rescue at right. All flies were at 3 days of age were were subjected in vials to vortex for 10 seconds immediately prior to the video shown.
(MOV)

S3 Video Flight assay of *pex2* mutant flies. Flies at 10 days of age were tapped gently into a clear column with a funnel at the top as shown. The flies have to fly from the center of the column to an edge and land. The *pex2*^*2*^ mutant flies almost all land at the bottom of the column, effectively flightless.
(MOV)

S4 Video Flight assay of *pex2* Rescue flies. Flies at 10 days of age were tapped gently into a clear column with a funnel at the top as shown. The flies have to fly from the center of the column to an edge and land. The *pex2*^*2*^ Rescue flies in contrast to the mutants almost all land at the top of the column.
(MOV)

S5 Video Flight assay of *pex16* mutant flies. Flies at 10 days of age were tapped gently into a clear column with a funnel at the top as shown. The flies have to fly from the center of the column to an edge and land. The *pex16*^*1*^ mutant flies with one exception almost all land at the bottom of the column, effectively flightless.
(MOV)

S6 Video Flight assay of *pex16* Rescue flies. Flies at 10 days of age were tapped gently into a clear column with a funnel at the top as shown. The flies have to fly from the center of the column to an edge and land. The *pex16*^*1*^ Rescue flies in contrast to the mutants almost all land at the top of the column.
(MOV)

S1 Table Analysis of peroxisomal lipids in fly and human samples. Source data from the GC MS analysis on fly and human samples for peroxisomal lipids as well as source data of LC-MSMS analysis
(XLSX)

S2 Table Metabolomic heat map of *Drosophila pex* mutants. Metabolite Heat Map a heat map comprised of ratios of analytes is shown for 347 individual metabolites. Columns include Pathway sort order, Super pathway, Sub pathway, Biochemical Name, Human Metabolome Database (HMDB) identifier, each genotype ratio, mutant/control or mutant to rescue is shown with indicators for significantly altered analytes, analytes narrowly missing statistical cutoffs with color codes for increased or decreased as indicated.
(XLSX)

S3 Table Metabolomic raw data of *Drosophila pex* mutants. Raw data from metabolomics measurements used to generate the ratios in S1 Table. Metabolites the metabolite measurements for each analyte and each pool (5 replicates of 100 flies each) are shown.
(XLSX)

S4 Table Consistently altered metabolites in *Drosophila pex* mutants. Altered Metabolites includes lists of consistently altered metabolites when considering significant or marginally significant changes (only considered if significant in the same direction). Lists were compiled for the *pex2* signature metabolites-common to *pex2*^*2*^*/pex2*^*2*^ Rescue and *pex2*^*1*^*/pex2*^*1*^ Rescue, the pex16 signature metabolites-common to *pex16*^*1*^*/pex16*^*1*^ Rescue and *pex16*^*EY*^*/pex16*^*EY*^ Rescue, and the null alleles-common to *pex2*^*2*^*/pex2*^*2*^ Rescue, *pex2*^*1*^*/pex2*^*1*^ Rescue, and *pex16*^*1*^*/pex16*^*1*^ Rescue.
(XLSX)

S5 Table Mitochondrial electron transport chain activity in *Drosophila pex2* mutants. Mitochondrial electron transport chain activity in isolated mitochondrial from adult flies. Source data is shown.
(XLSX)

S6 Table *Drosophila* peroxisomal gene clusters. The gene clusters for *Drosophila* peroxisomal genes- the top 1000 co-regulated genes in the fly genome are shown for each of the clusters 5 clusters for *Drosophila* data (See Materials and Methods).
(XLSX)

S7 Table Mouse peroxisomal gene clusters. The gene clusters for mouse peroxisomal genes- the top 1000 co-regulated genes in the fly genome are shown for each of the clusters,4 clusters for mouse liver. (See Materials and Methods)
(XLSX)

S8 Table Overview of representation of PGC and AMPK regulated genes in peroxisomal gene clusters. PGC and AMPK- highlight genes represented in the top 1000 co-regulated genes from the AMPK pathway and PGC pathway showing those which are common between mouse liver and *Drosophila*.
(XLSX)
