# Supplementary figures and images for: Peroxisomal biogenesis is genetically and biochemically linked to carbohydrate metabolism in Drosophila and mouse
Source: PLoS Genet. 2017 Jun 22;13(6):e1006825. doi: 10.1371/journal.pgen.1006825 (PMC5480855; doi:10.1371/journal.pgen.1006825)

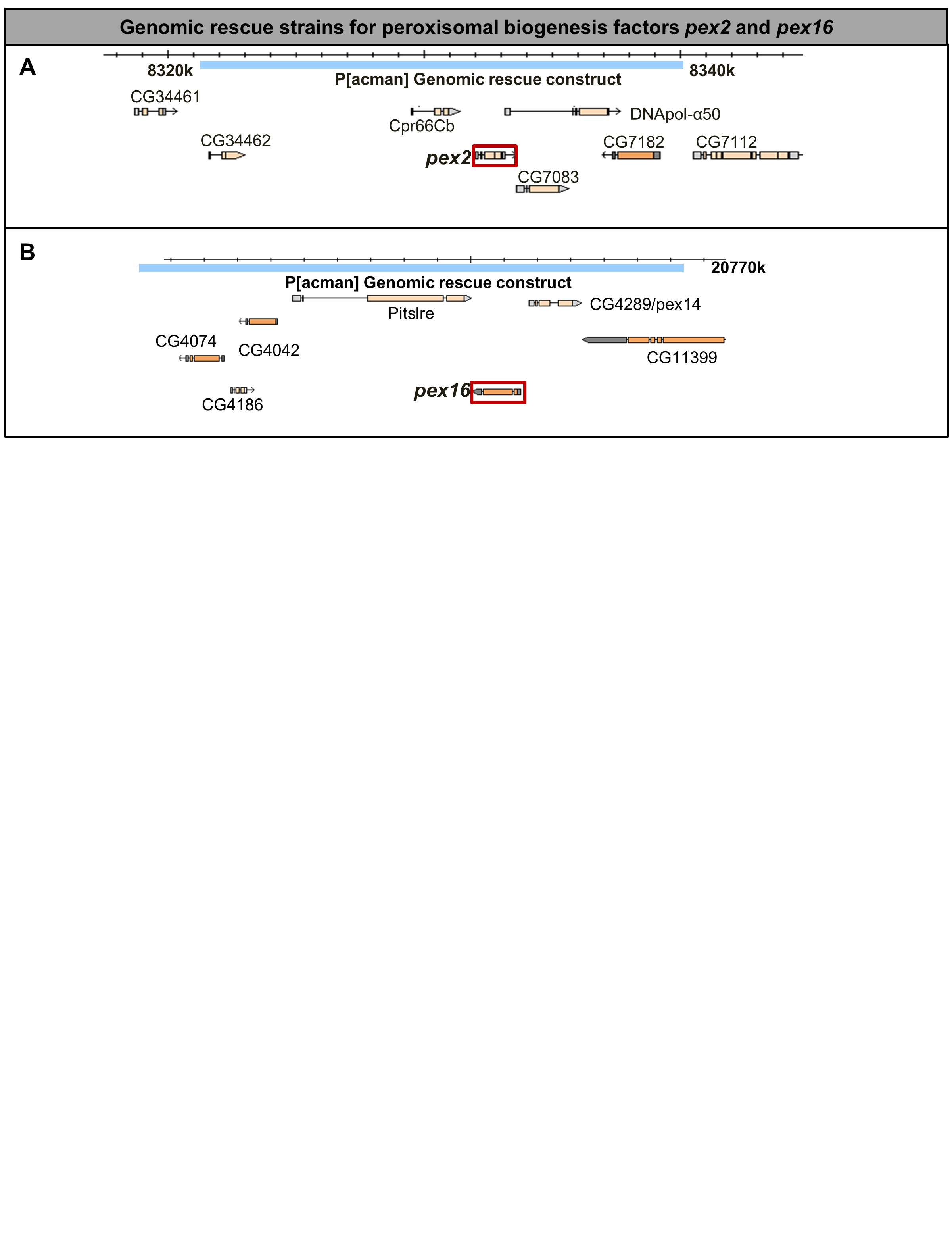

Supplement: S1 Fig — (A) The pex2 gene is shown in genomic context, the blue bar represents the specific genomic rescue construct which was used to produce a transgenic line for rescue experiments. A red box indicates the exons shown in Fig 1A.(B) The pex16 gene is shown in genomic context, the blue bar represents the specific genomic rescue construct which was used to produce a transgenic line for rescue experiments. A red box indicates the exons shown in Fig 1B. (TIF) [file pgen.1006825.s001.tif]

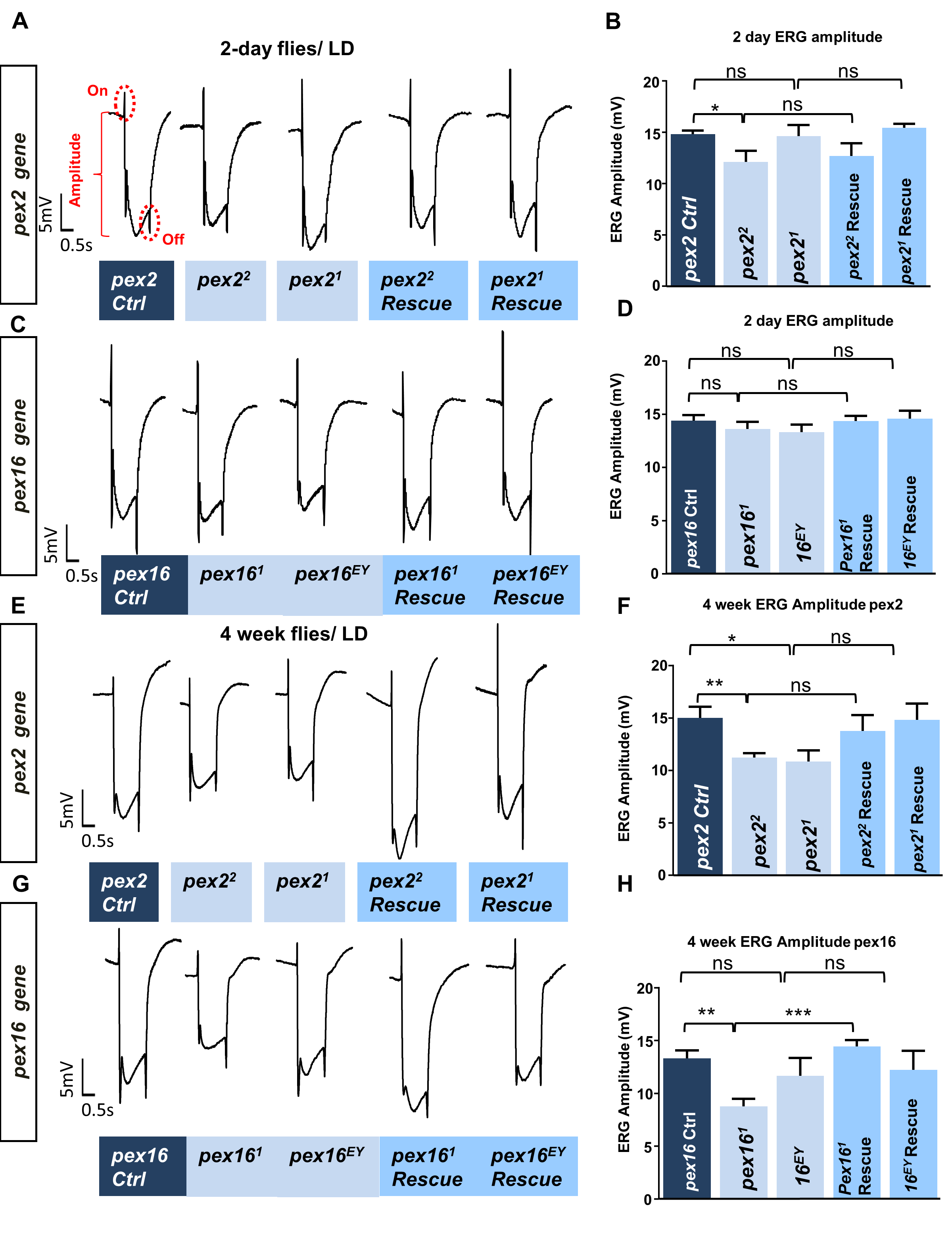

Supplement: S2 Fig — (A) Electroretingrams demonstrate the field potential after light exposure in photoreceptors, the amplitude of depolarization (red bracket) was assessed after the “on” and before the “off” transient indicating synaptic activity. The pex2 tracings show no differences between mutant and rescue animal for the indicated genotypes(B) Quantification of the amplitude for 2-day pex2 flies student’s t-test, P>0.05 = ns, P< 0.05 = *, P<0.01 = **, P<0.001 = ***.(C) The pex16 tracings show no differences between mutant and rescue animal for the indicated genotypes(D) Quantification of the amplitude for 2-day pex16 flies student’s t-test, P>0.05 = ns, P< 0.05 = *, P<0.01 = **, P<0.001 = ***.(E) The pex2 tracings show reduced amplitude in the mutants between mutant and rescue animals at 4 weeks after 12 hour light-dark cycle.(F) Quantification of the amplitude for 4-week pex2 flies showing a statistically significant reduction in amplitude in the mutants.(G) The pex16 tracings show reduced amplitude in the mutants between mutant and rescue animals at 4 weeks for the pex161 allele but not for the pex16EY.(H) Quantification of the amplitude for 4-week pex16-1 flies showing a statistically significant reduction in amplitude in the mutants. (TIF) [file pgen.1006825.s002.tif]

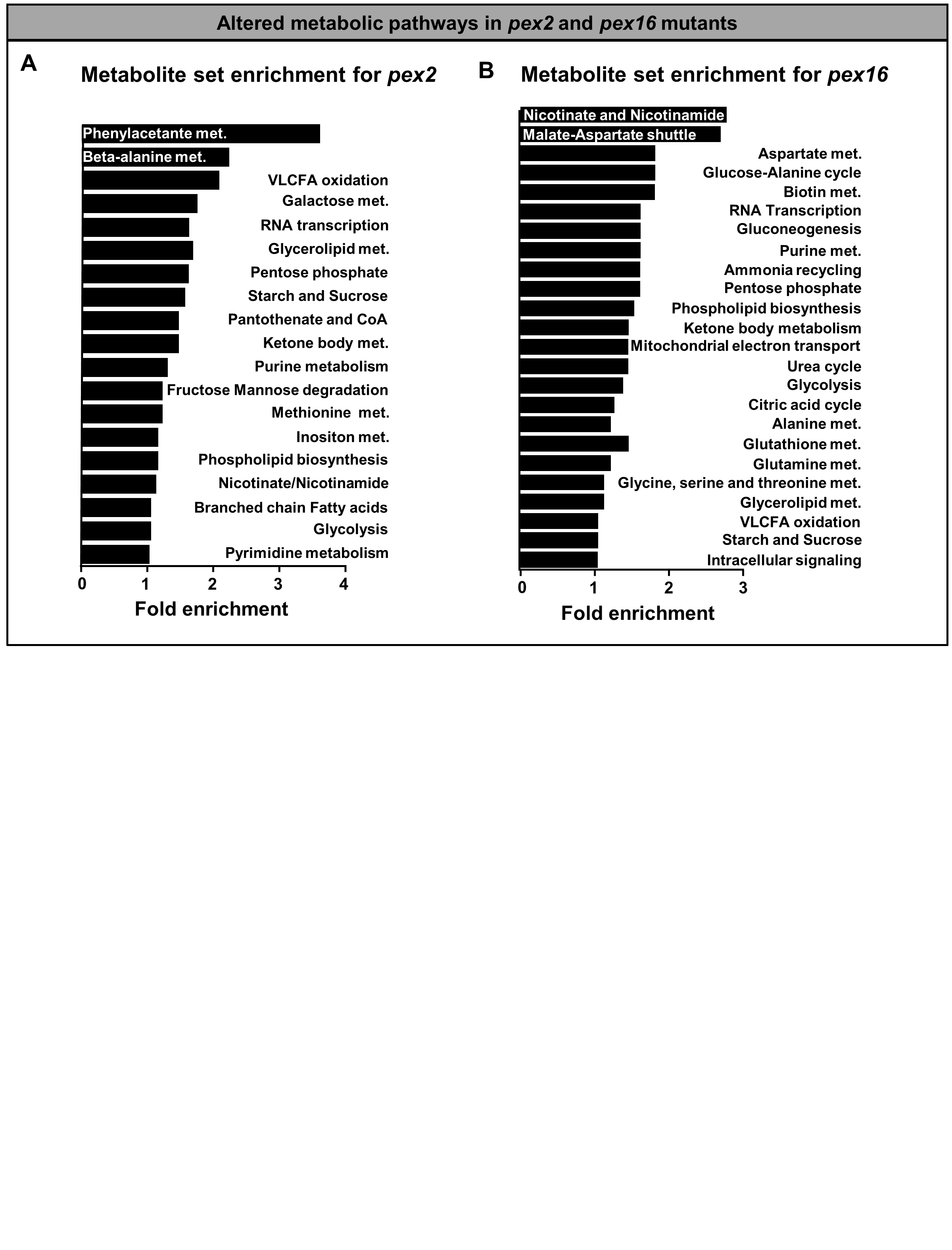

Supplement: S3 Fig — (A) Metabolite set enrichment fold enrichment was performed on the subset of metabolites that were consistently altered in pex2 both deletion alleles. The fold enrichment values are shown.(B) Metabolite set enrichment fold enrichment was performed on the subset of metabolites that were consistently altered in pex16 deletion allele. The fold enrichment values are shown. (TIF) [file pgen.1006825.s003.tif]

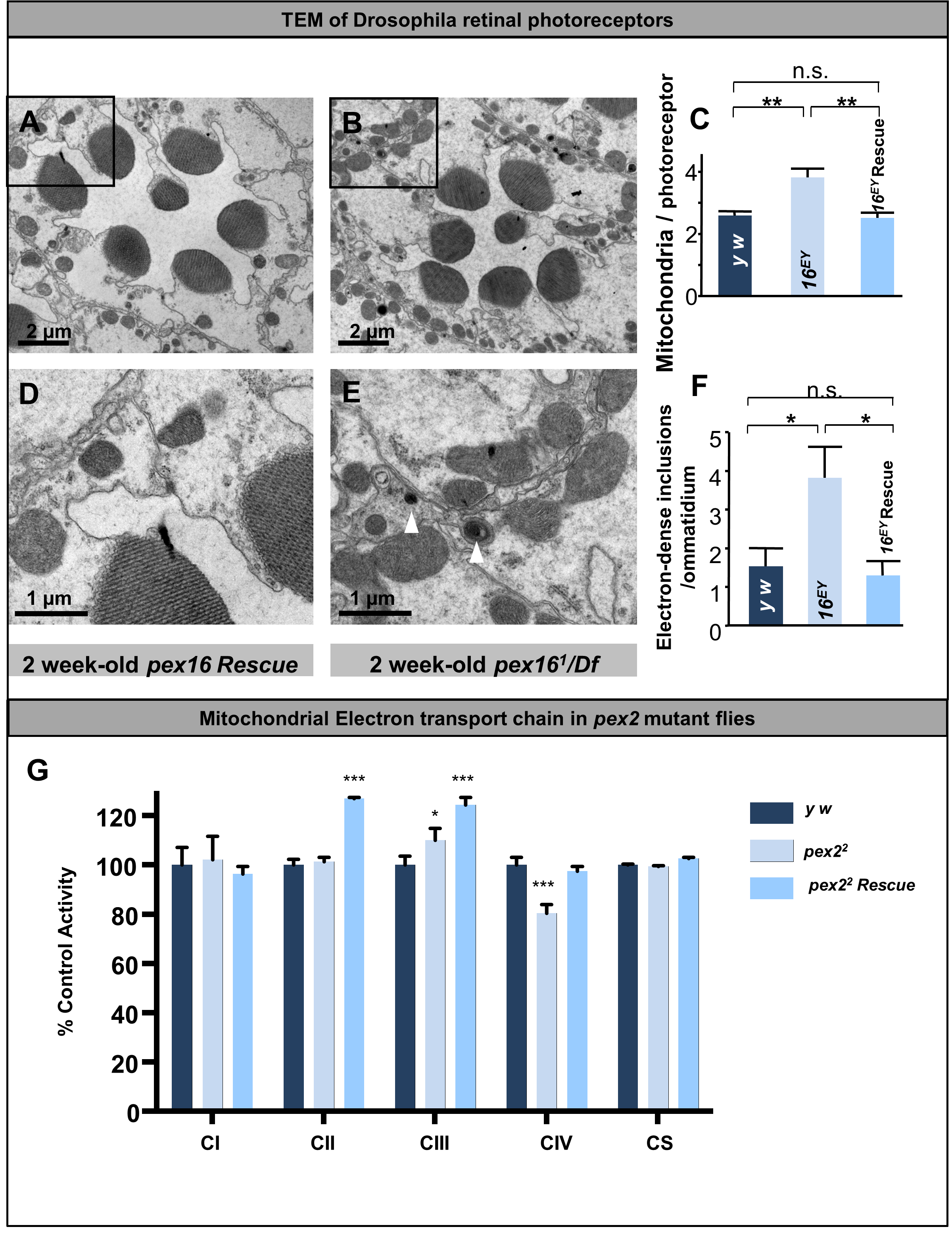

Supplement: S4 Fig — (A) Transmission electron microscopy (TEM) of Drosophila photoreceptors. Normal ultrastructure of the photoreceptors in the retina in 2 week old pex16EY Rescue animals with seven photoreceptors, the dark rhabdomeres and the mitochondria which often cluster in the cell body of the photoreceptor.(B) TEM of pex16EY animals showing apparent increase in the number of mitochondria per photoreceptor terminal.(C) Quantification of mitochondria per photoreceptor.(D) Inset of A showing mitochondria in the photoreceptor(E) Inset of B showing mitochondria and electron dense inclusions.(F) Quantification of E.(G) Mitochondrial electron transport chain activity in the pex2 mutants. Stars indicate activities with statistically significant differences from the control activity. (TIF) [file pgen.1006825.s004.tif]

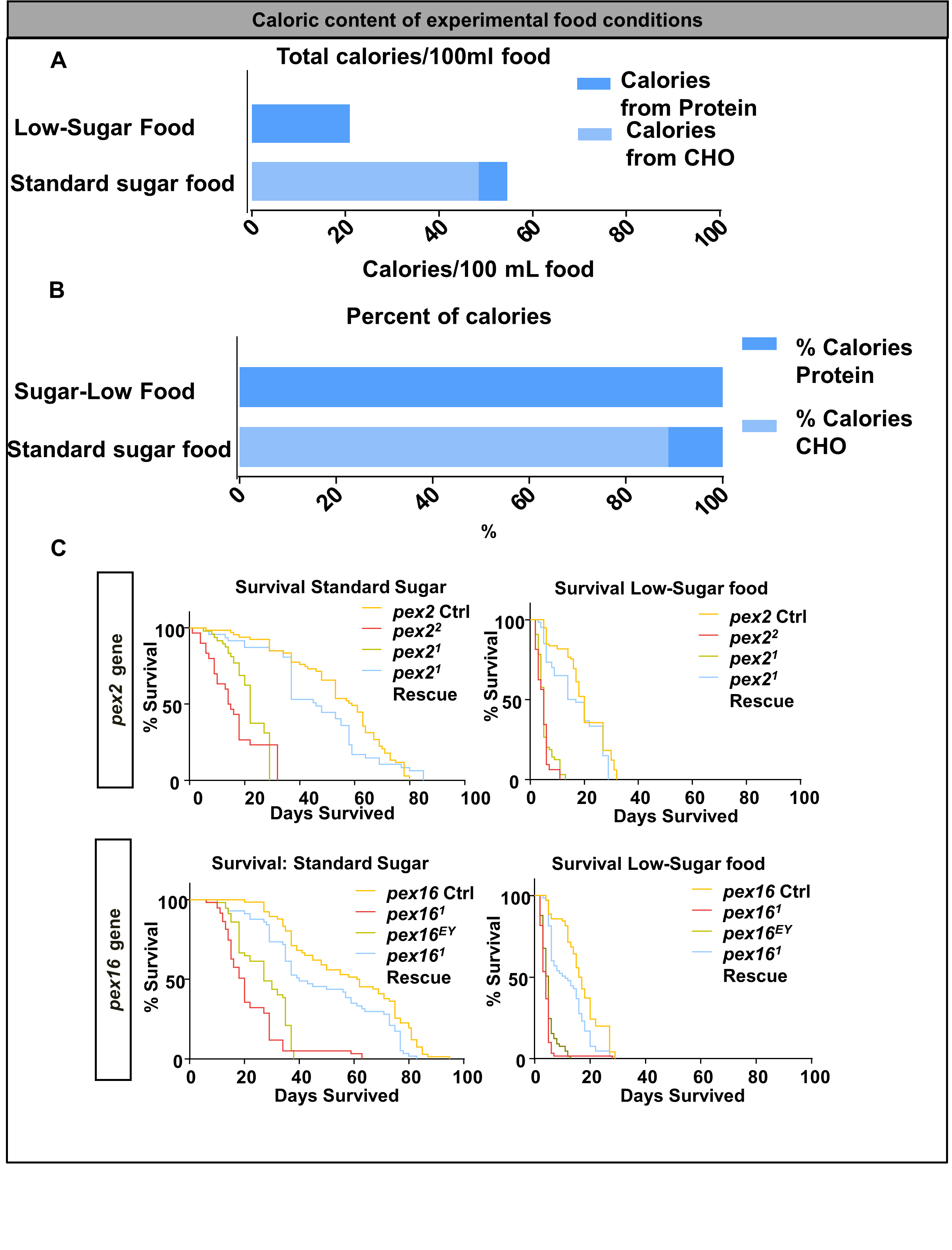

Supplement: S5 Fig — (A) Composition of the food for the conditional food experiments. Total calories per 100 mL of food is shown.(B) Percent of calories for conditional food.(C) Kaplan-Meier curves for the quantification shown in Fig 8B and 8C. (TIF) [file pgen.1006825.s005.tif]

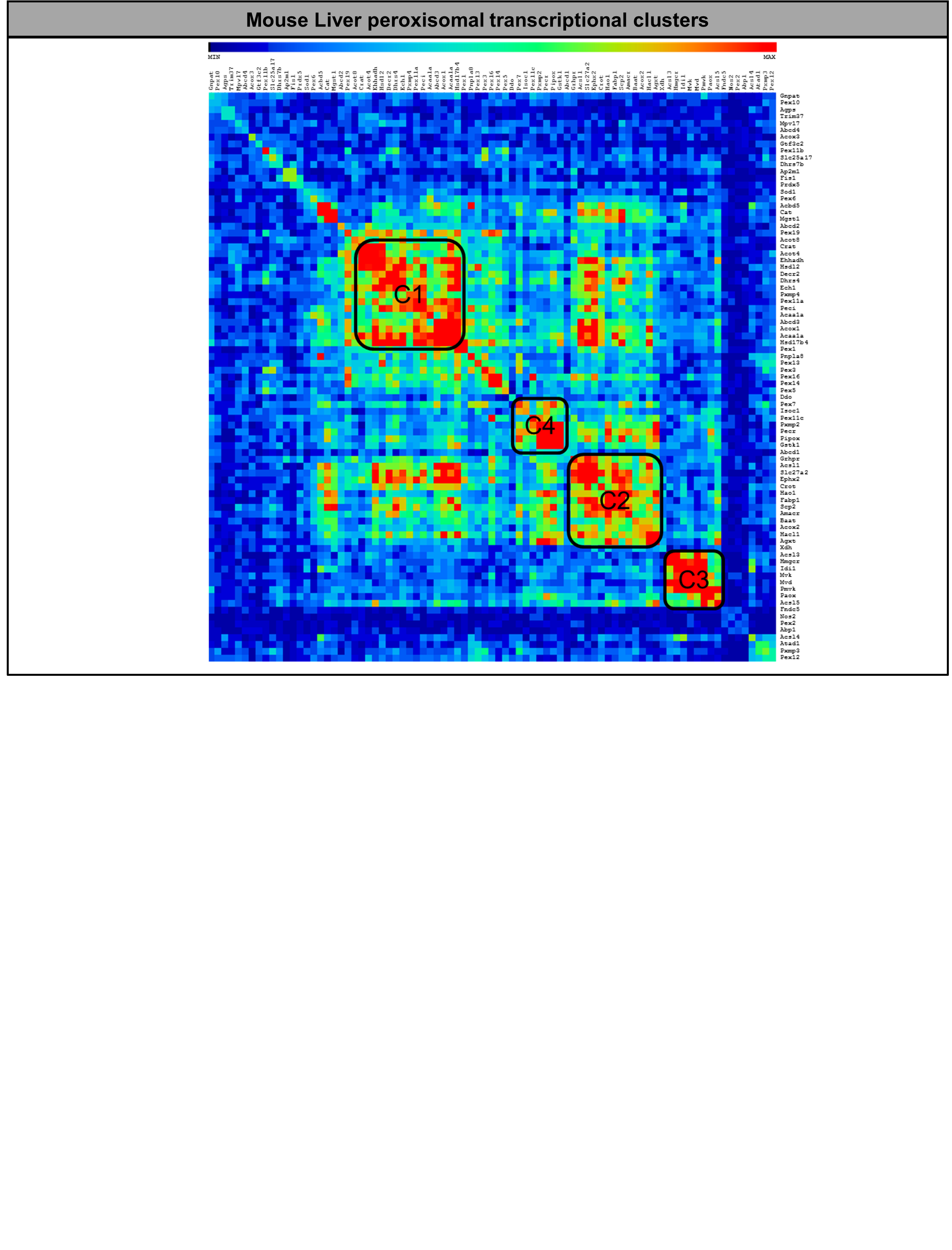

Supplement: S6 Fig — Mouse liver peroxisomal gene clusters, mouse liver peroxisomal genes are grouped into 4 closely co-regulated clusters. (TIF) [file pgen.1006825.s006.tif]

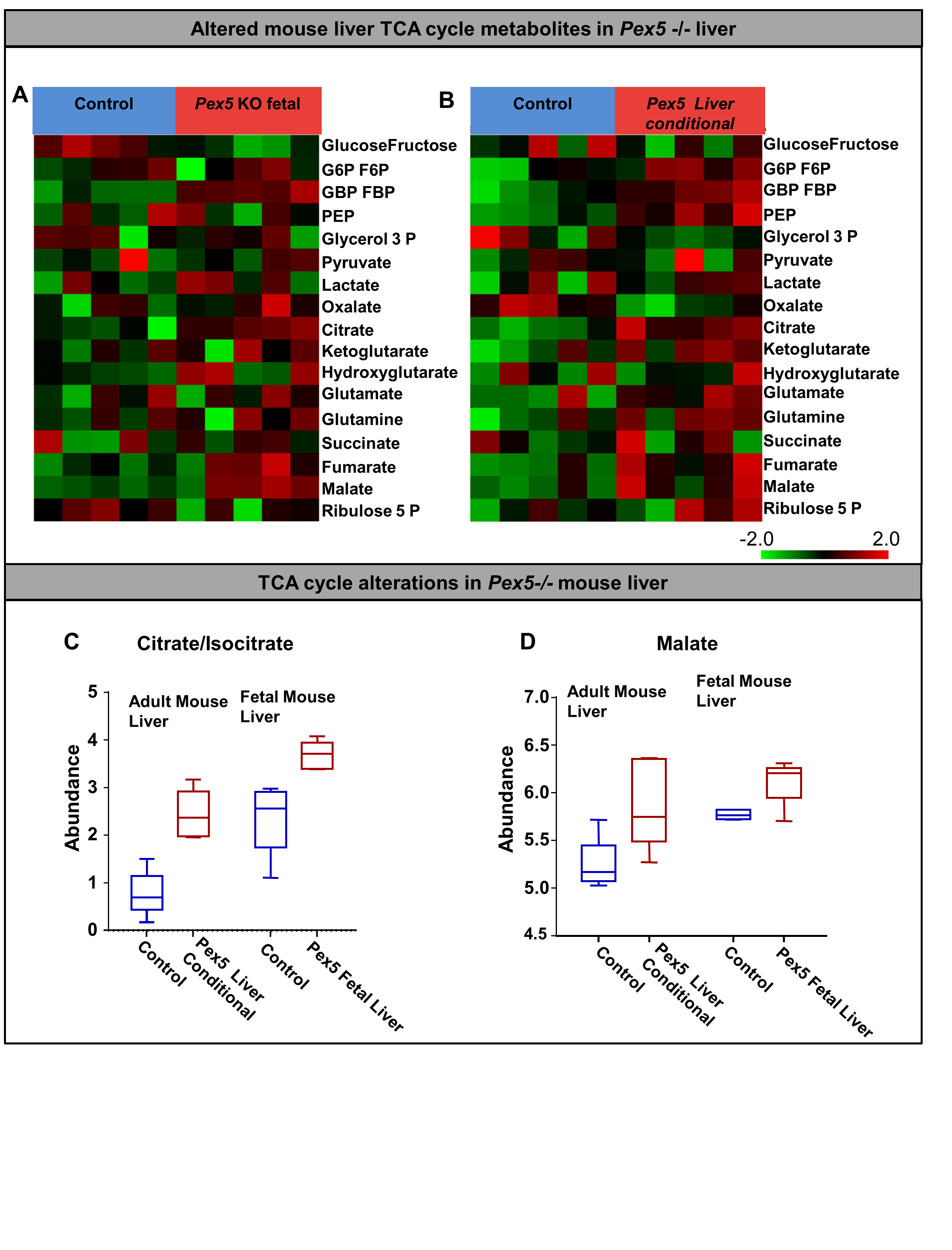

Supplement: S7 Fig — (A) Heat map of Pex5 knockout mice versus controls showing some alterations in citrate and malate(B) Heat map of Pex5 liver conditional mice versus controls showing a number of altered analytes including G6P/F6P, citrate, ketoglutarate, glutamate, fumarate and malate.(C) Abundance of Citrate/Isocitrate in Targeted metabolomics in adult and fetal mouse liver showing increased abundance in both global and conditional Pex5 murine liver compared to controls.(D) Abundance of Malate in Targeted metabolomics in adult and fetal mouse liver showing increased abundance in both global and conditional Pex5 murine liver compared to controls. (TIF) [file pgen.1006825.s007.tif]
